# Microwave-Assisted Sulfuric Acid Leaching of Vanadium from Pre-Decalcified Vanadium-Bearing Steel Slag

**DOI:** 10.3390/molecules31183330

**Published:** 2026-09-19

**Authors:** Minhao Zhang, Zekun Wang, Ang Zhao, Xuan Liu, Le Wang, Jinglong Liang

**Affiliations:** 1 College of Metallurgy and Energy, North China University of Science and Technology, Tangshan 063210, China; zhangminhao1415@163.com (M.Z.); wangzekun2333@163.com (Z.W.); 15110759912@163.com (A.Z.); 15903427492@163.com (X.L.); ljl@ncst.edu.cn (J.L.); 2 Yanzhao Iron and Steel Laboratory, North China University of Science and Technology, 21 Bohai Street, Tangshan 063210, China

**Keywords:** vanadium-bearing steel slag, pre-decalcification, microwave-assisted sulfuric acid leaching, kinetics

## Abstract

Vanadium-bearing steel slag has attracted considerable attention as a secondary source of vanadium. Pre-decalcified vanadium-bearing steel slag served as the feedstock to evaluate vanadium leaching under microwave irradiation and conventional water-bath heating by varying sulfuric acid concentration, temperature, leaching time, and liquid-to-solid ratio. The leaching performance was further evaluated through countercurrent leaching experiments, and kinetic analysis was conducted to investigate the rate-controlling behavior. The results demonstrated that the optimum leaching conditions under microwave irradiation were a liquid-to-solid ratio of 8 mL·g^−1^, a leaching time of 60 min, a sulfuric acid concentration of 15%, and a temperature of 70 °C, yielding a vanadium leaching rate of 74.43%. Multistage leachate reuse increased the normalized V enrichment index from 74.43% after the initial single-stage leaching to 89.93% after two reuse stages, with only a limited further increase thereafter. Kinetic analysis indicated a constant-particle-size shrinking-core mechanism for vanadium leaching. Sulfuric acid concentration exhibited an apparent reaction order of 1.80, and the apparent activation energy was 57.20 kJ·mol^−1^. Internal diffusion was identified as the predominant rate-controlling step, while the contribution of the interfacial chemical reaction could not be neglected. Microwave irradiation enhanced vanadium extraction through volumetric and selective heating effects, potentially promoting structural disruption of the pre-decalcified slag and facilitating leaching agent diffusion into the particle interior. These findings provide an effective hydrometallurgical strategy for sustainable vanadium recovery from industrial solid waste.

## 1. Introduction

Vanadium rarely occurs in economically exploitable independent deposits [1,2] and is mainly associated with titanomagnetite, stone coal, and other mineral resources [3,4]. Because it markedly improves the strength, ductility, and corrosion resistance of metallic materials, vanadium is widely used in steelmaking, energy storage, and catalysis and is therefore regarded as an important strategic metal resource.

During titanomagnetite smelting, vanadium-bearing hot metal is produced by high-temperature reduction, after which part of the vanadium is oxidized into the slag during converter blowing. During basic oxygen steelmaking, oxygen is introduced into the molten metal to remove carbon and other easily oxidized elements, while Ca-rich fluxes are added to promote slag formation. The resulting vanadium-bearing steel slag is a complex multiphase secondary resource with intricate mineral associations and strong elemental intergrowth. Its mineral assemblage is generally dominated by Ca- and Fe-bearing phases, including silicate phases such as larnite, ferrite phases such as brownmillerite, and Fe-rich oxide phases. Vanadium commonly occurs in these Ca- and Fe-bearing phases through isomorphous substitution or solid-solution incorporation rather than as an independent vanadium mineral, whereas Ca and Fe are the dominant constituents [5]. Therefore, selective separation of vanadium from the abundant matrix components is essential for its efficient valorization.

Vanadium is mainly recovered from such slag by roasting-leaching or direct leaching. Sodium roasting-leaching is widely applied because of its technological maturity, high conversion efficiency, and industrial adaptability [6]. During roasting, low-valence vanadium is oxidized into soluble vanadates, and phase evolution strongly affects subsequent recovery. Li et al. [7] showed that stepwise sodium roasting promoted selective oxidation of vanadium spinel into soluble sodium vanadate while chromium spinel remained comparatively stable. Ji et al. [8] further enhanced vanadate formation through a three-phase sodium roasting system that strengthened interfacial reactions at lower temperatures. However, salt-containing off-gas and high-salinity wastewater create substantial environmental burdens, and air pollution has become a major limitation to further application [9].

Calcification roasting is another established route for vanadium recovery [10]. Wen et al. [11] demonstrated that CaO accelerated spinel oxidation and promoted calcium vanadate formation in high-Cr vanadium slag. Compared with sodium roasting, this route causes less equipment corrosion and may offer energy-saving advantages. Nevertheless, stable Ca-V phases can hinder subsequent leaching, while the high consumption of calcium-containing additives increases material demand and limits large-scale industrial application.

Direct acid leaching has therefore attracted attention as a lower-temperature alternative. Vanadium is dissolved in sulfuric acid and subsequently recovered by solvent extraction and ammonium salt precipitation. Zhang et al. [12] showed that direct acid leaching can avoid the high energy consumption and gaseous emissions associated with roasting. However, Mg, Ca, Fe, Cr also dissolve readily, increasing acid consumption, contaminating the leachate, and complicating downstream vanadium separation and purification. Effective pretreatment and process intensification are therefore needed to improve selectivity.

Microwave heating offers volumetric, rapid, and selective energy delivery [13]. Previous work showed that, under identical 20 min conditions, microwave irradiation increased vanadium leaching from 46% to 96% and reduced the apparent activation energy from 40.5 to 14.8 kJ·mol^−1^ [14]. This indicates lower diffusion resistance and enhanced activation of vanadium-bearing phases. Omran et al. [15] further reported that selective microwave absorption by iron-bearing phases accelerated reactions in metallurgical wastes, disrupted compact structures, shortened heating time, and reduced energy consumption. These characteristics make microwave heating promising for low-temperature intensification of slag leaching.

Countercurrent leaching can maintain a high concentration gradient through multistage circulation, thereby improving mass transfer and reagent utilization. Zhu et al. [16] achieved 86% vanadium recovery from stone coal using a three-stage sulfuric acid system and reported lower acid consumption and a higher leachate pH than single-stage leaching. Kologrieva et al. [17] developed a three-stage process for vanadium-bearing hydrometallurgical residues that reduced liquid consumption and produced a concentrate containing approximately 70 wt.% V_2_O_5_. These results confirm the potential of countercurrent operation for efficient vanadium extraction, solution enrichment, and subsequent recovery.

Motivated by the aforementioned merits of microwave-assisted heating and countercurrent leaching, the objective of this work is to develop a green and efficient microwave-driven sulfuric acid leaching approach for recovering vanadium from pre-decalcified vanadium-bearing steel slag. The effects of key operating parameters on vanadium leaching were systematically evaluated under microwave irradiation and compared with those obtained under conventional water-bath heating. The process variables were optimized to maximize vanadium recovery. Furthermore, the leaching kinetics under microwave irradiation were investigated to determine the rate-limiting step and elucidate the factors controlling vanadium dissolution dynamics. The findings offer theoretical underpinnings and practical implications for the efficient extraction of vanadium from vanadium-bearing steel slag.

## 2. Results and Discussion

### 2.1. Thermodynamic Analysis

**Figure 1:**
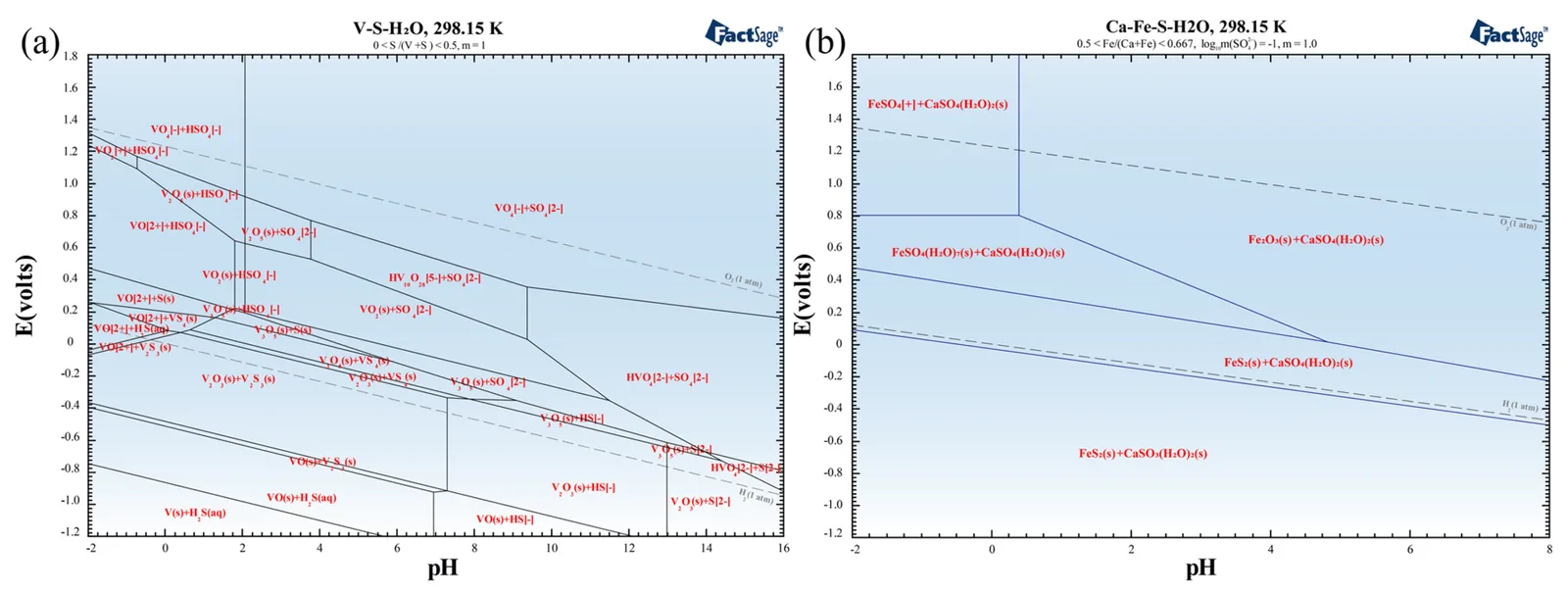
E-pH diagrams of different systems: (**a**) V-S-H_2_O system; (**b**) Ca-Fe-S-H_2_O system.

Thermodynamic analysis of the vanadium leaching system was carried out using FactSage 6.4, and the E-pH diagrams of the V-S-H_2_O and Ca-Fe-S-H_2_O systems at 298.15 K are presented in Figure 1. As shown in Figure 1a, both V^4+^ and V^5+^ aqueous species can be thermodynamically stable under acidic conditions, depending on the redox potential. V^4+^ is mainly represented by VO^2+^, whereas V^5+^ can exist as VO_2_^+^ under sufficiently oxidizing conditions. Vanadium species may also coexist with SO_4_^2−^ and HSO_4_^−^ in the acidic sulfate medium, indicating that vanadium can remain in soluble forms over a relatively wide potential range. Therefore, the acidic leaching system used in this study is thermodynamically favorable for maintaining dissolved vanadium species and promoting vanadium dissolution.

To further consider the behavior of the Ca- and Fe-bearing components associated with the dicalcium ferrite phase, the E-pH diagram of the Ca-Fe-S-H_2_O system was constructed, as shown in Figure 1b. Under acidic sulfate conditions, Ca tends to occur as stable calcium sulfate-containing solid phases, while the stable forms of Fe vary with the redox potential. This result indicates that, following acid-induced decomposition of Ca-Fe-bearing phases, Ca can be stabilized in the form of calcium sulfate, whereas Fe may remain in sulfate-containing or oxide-bearing forms depending on the solution conditions.

**Figure 2:**
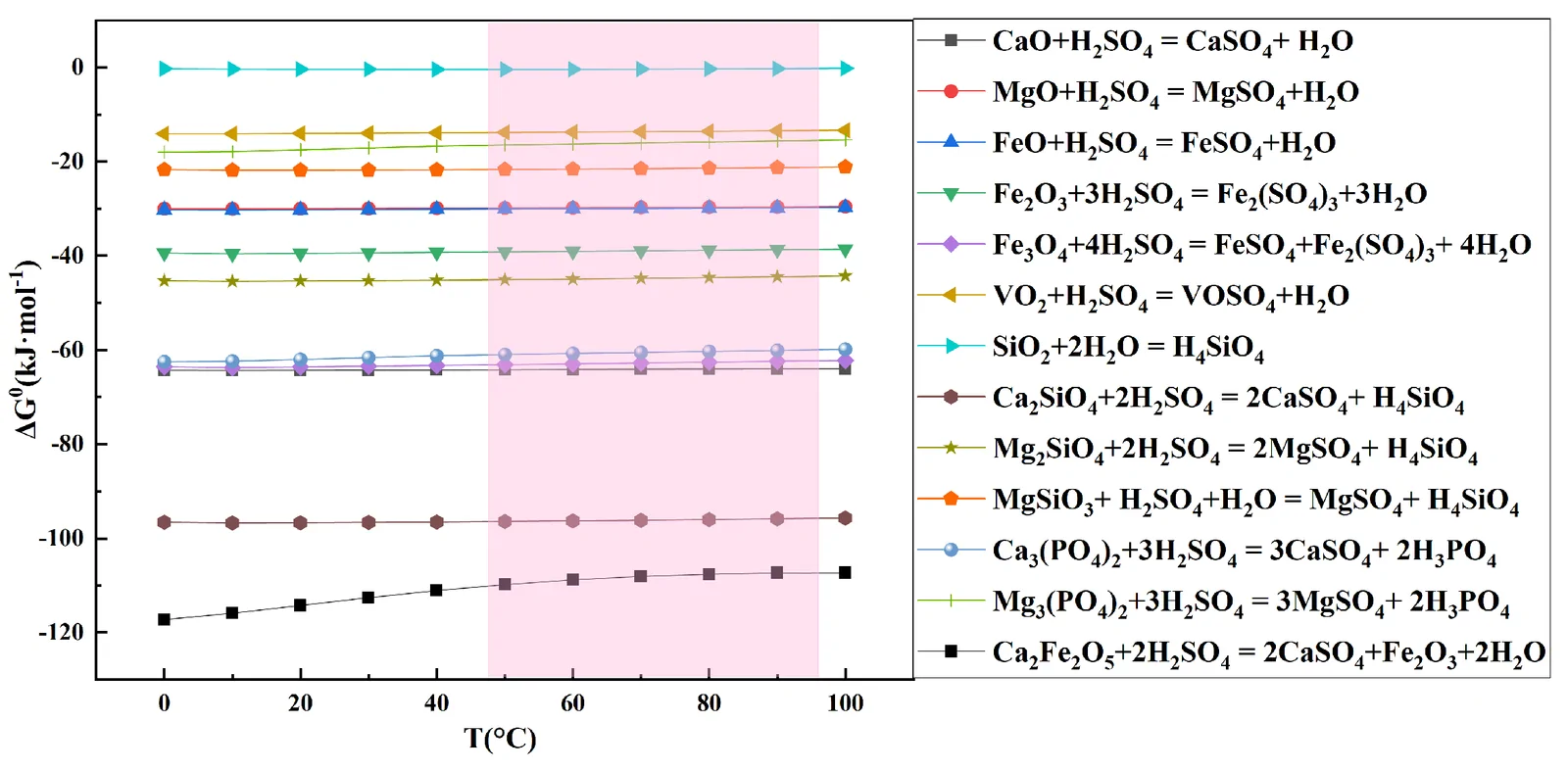
Δ*G*^0^ of relevant reactions as a function of temperature.

Furthermore, Δ*G* of the relevant reactions in the sulfuric acid leaching system were evaluated using HSC Chemistry 6.0, and the results are presented in Figure 2. The calculated Δ*G*^0^ values of the considered reactions are negative within the investigated temperature range, indicating that the dissolution or transformation of the major mineral components is thermodynamically feasible. In particular, the reaction of dicalcium ferrite, an important V-bearing phase in the pre-decalcified slag, with sulfuric acid was additionally considered. The Δ*G*^0^ value of this reaction remains negative throughout the investigated temperature range, demonstrating that the acid-induced decomposition of Ca_2_Fe_2_O_5_ is thermodynamically favorable. During this process, Ca is converted into calcium sulfate, while decomposition of the ferrite structure can facilitate the release of vanadium associated with this phase.

### 2.2. Single-Factor Vanadium Extraction Experiments

Vanadium leaching rate was employed as the key evaluation index in the present work. The influence of four critical process variables, including L/S ratio, leaching time, leaching temperature, and sulfuric acid concentration, on vanadium leaching behavior was systematically examined to determine their influencing laws and optimal ranges. All leaching tests were conducted in triplicate, and the reported values are arithmetic means of three independent experiments. Error bars for vanadium leaching rates denote one standard deviation (SD, *n* = 3), reflecting the consistency and reproducibility of the experimental data. Results for impurity elements are presented as mean values only to show their leaching trends.

#### 2.2.1. Effect of Sulfuric Acid Concentration on Vanadium Leaching

**Figure 3:**
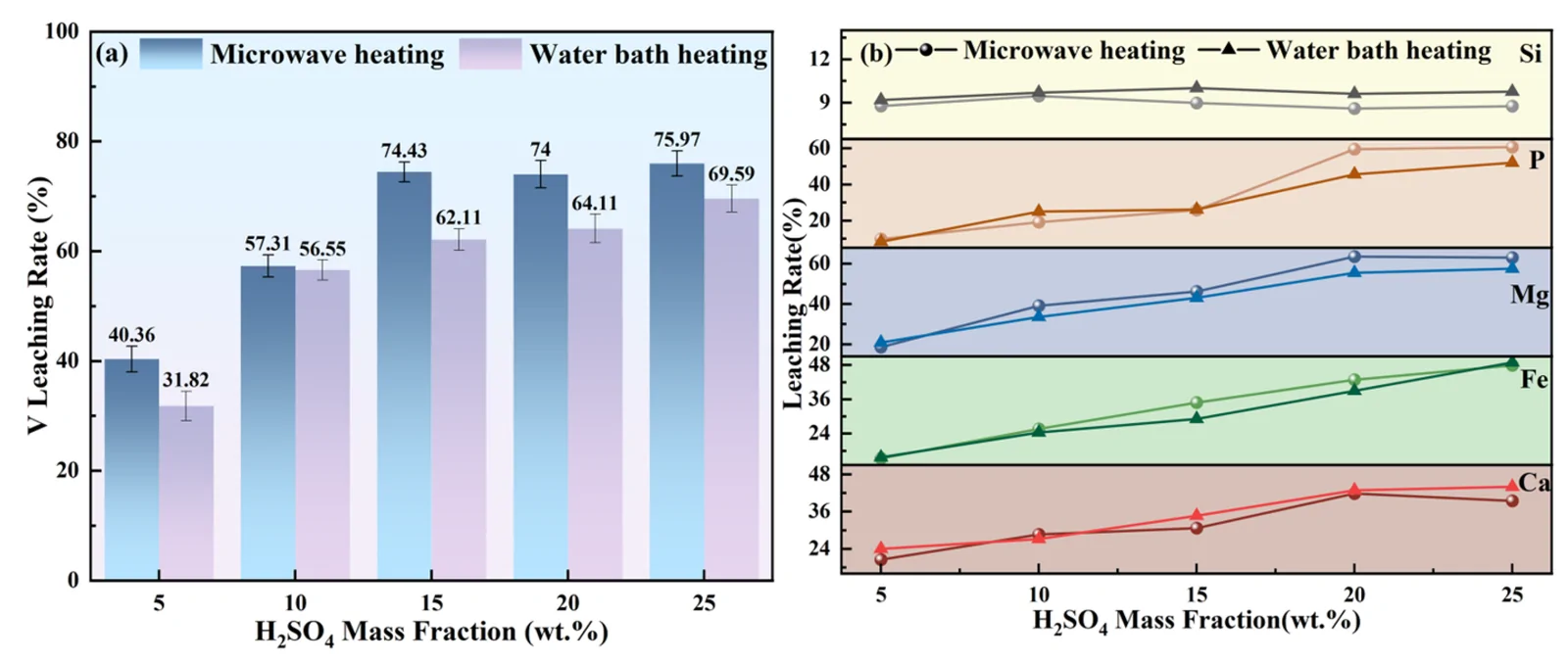
Effect of sulfuric acid concentration on the leaching rates of various elements under different heating methods: (**a**) vanadium leaching rate; (**b**) other elements leaching rates (temperature: 70 °C, time: 60 min, liquid-to-solid ratio: 8 mL·g^−1^).

Sulfuric acid concentration is a key parameter governing the hydrometallurgical extraction of vanadium-bearing steel slag. It not only determines the acidity and reaction driving force of the system but also affects the dissolution behavior of the pre-decalcified slag by regulating the activity of H^+^ in solution. Combined with the E-pH thermodynamic analysis of the V-S-H_2_O system, a strongly acidic environment is essential for maintaining vanadium in a stable ionic form. Under fixed conditions of an L/S ratio of 8 mL·g^−1^,70 °C, and 60 min, the influence of sulfuric acid concentration (5–25 wt.%, in 5 wt.% increments) on vanadium leaching was investigated, as shown in Figure 3.

Figure 3a illustrates the vanadium leaching performance under different heating methods. The data reveal that the vanadium extraction initially rises before leveling off with increasing sulfuric acid concentration. When the acid concentration is below 15 wt.%, insufficient H^+^ in the system limits the destruction of the crystalline structure of the pre-decalcified slag. Meanwhile, residual alkaline components such as calcium consume acid, resulting in incomplete dissolution and thus low vanadium recovery. When the sulfuric acid concentration increases to 15 wt.%, the reaction between H^+^ and vanadium-bearing phases is significantly enhanced, leading to extensive lattice destruction and vanadium release as soluble ionic species into solution. Under microwave heating, the vanadium leaching rate reaches a maximum of 74.43%. Further increasing acid concentration does not significantly improve leaching performance, indicating that the system has approached thermodynamic equilibrium. Under conventional water-bath heating, the vanadium leaching rate remains consistently lower than that under microwave conditions at the same acid concentration, demonstrating the superiority of microwave-assisted heating.

**Figure 4:**
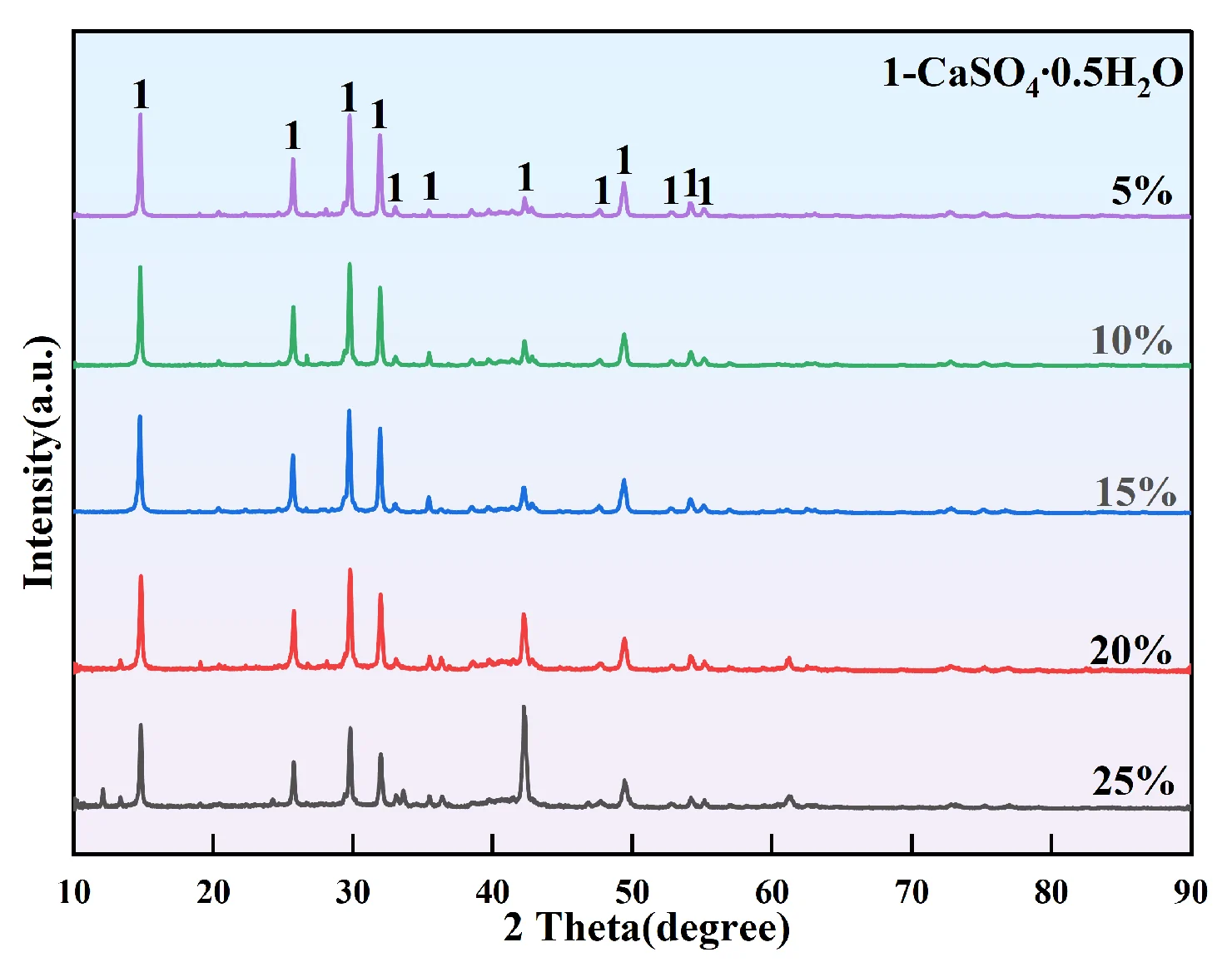
XRD patterns of leaching residues obtained at different sulfuric acid concentrations under microwave irradiation.

Figure 3b shows the leaching trends of impurity elements under different acid concentrations. Silicon remains relatively stable, while Ca, Fe, Mg, and P all exhibit increasing trends with increasing acid concentration. Figure 4 displays XRD patterns of leaching residues under microwave heating at varying sulfuric acid levels. The results indicate that the residues are mainly composed of calcium sulfate hemihydrate (CaSO_4_·0.5H_2_O), which is formed due to the precipitation reaction between Ca^2+^ and SO_4_^2−^ in a strongly acidic sulfate system and is thermodynamically stable under these conditions. No obvious diffraction peaks attributable to C_2_F and FeO were detected, suggesting that these dominant mineral phases in the pre-decalcified slag were substantially decomposed during acid leaching, thereby releasing the encapsulated vanadium into solution.

Unlike conventional heating, microwave irradiation exhibits both volumetric and selective heating characteristics [18]. In a microwave field, polar species such as water and sulfuric acid molecules absorb microwave energy through dipolar polarization and ionic conduction mechanisms, enabling volumetric and rapid heating of the system. In addition, different mineral phases exhibit distinct dielectric loss factors. Iron- and vanadium-bearing phases generally show higher microwave absorption capacity [19], which may result in localized temperature gradients and thermal stresses and could promote microstructural disruption, thereby facilitating the exposure and dissolution of vanadium-bearing phases. Therefore, this effect is proposed as a possible mechanism for the enhanced vanadium leaching observed under microwave irradiation.

Insufficient sulfuric acid concentration results in limited H^+^ availability and low vanadium leaching rate. In contrast, excessively high acid concentration increases reagent consumption, equipment corrosion risk, and impurity dissolution, thereby increasing downstream neutralization and purification burden. Considering vanadium recovery efficiency, economic feasibility, and impurity control, 15 wt.% sulfuric acid under microwave heating is identified as the optimal condition.

#### 2.2.2. Effect of Leaching Temperature on Vanadium Leaching

**Figure 5:**
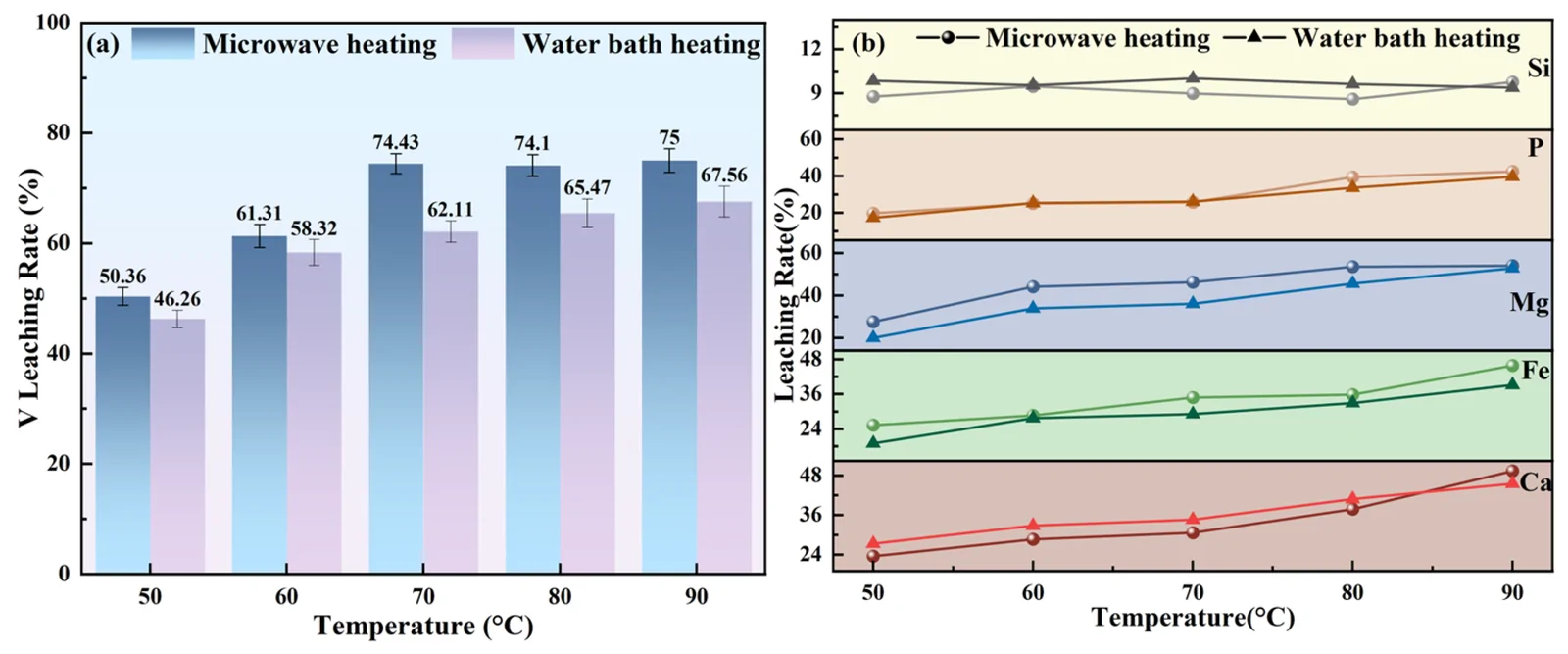
Effect of leaching temperature on the leaching rates of various elements under different heating methods: (**a**) vanadium leaching rate; (**b**) other elements leaching rates (sulfuric acid concentration: 15 wt.%, time: 60 min, liquid-to-solid ratio: 8 mL·g^−1^).

The leaching temperature influences vanadium extraction by affecting molecular motion and ion diffusion kinetics in the pre-decalcified slag. Under fixed conditions of an L/S ratio of 8 mL·g^−1^, a leaching time of 60 min, and 15 wt.% sulfuric acid concentration, the effect of temperature (50–90 °C, at 10 °C intervals) on vanadium leaching behavior was investigated, as shown in Figure 5.

Generally, increasing temperature accelerates both interfacial reaction kinetics and mass transfer processes. As illustrated in Figure 5a, microwave-assisted vanadium extraction rises markedly as temperature increases to 70 °C. This can be attributed to the relatively low thermal energy at lower temperatures, where molecular motion in solution is limited and the interaction of V-bearing mineral phases with sulfuric acid proceeds slowly. As temperature increases, enhanced reaction kinetics and diffusion rates facilitate the exposure of previously unreacted active sites within the mineral matrix, thereby promoting vanadium dissolution.

At 70 °C, the vanadium leaching rate reaches a maximum of 74.43%. Further temperature increase does not significantly enhance vanadium extraction but instead promotes excessive dissolution of impurity elements (Figure 5b), thereby reducing leachate selectivity.

Under conventional water-bath heating, a noticeable increase in vanadium leaching is observed up to 60 °C. Beyond this point, the leaching rate increases only slightly, peaking at 67.56% at 90 °C, which is significantly lower than that achieved under microwave irradiation at 70 °C. This difference indicates that microwave irradiation provides an additional enhancement pathway beyond conventional thermal activation.

This enhancement can be attributed to the unique energy transfer mechanism of microwave heating, which enables rapid volumetric heating and may induce localized thermal gradients within the heterogeneous slag matrix. Such effects can facilitate stress accumulation and microcrack formation, thereby weakening the structural integrity of vanadium-bearing phases and improving their accessibility to the leaching agent. In contrast, conventional heating relies primarily on external conductive heat transfer, resulting in slower and less efficient activation of the solid matrix.

Moreover, microwave irradiation not only enhances thermal activation but also promotes structural disordering and microfracturing, leading to more efficient liberation of vanadium-bearing phases embedded in calcium- and iron-rich mineral matrices.

**Figure 6:**
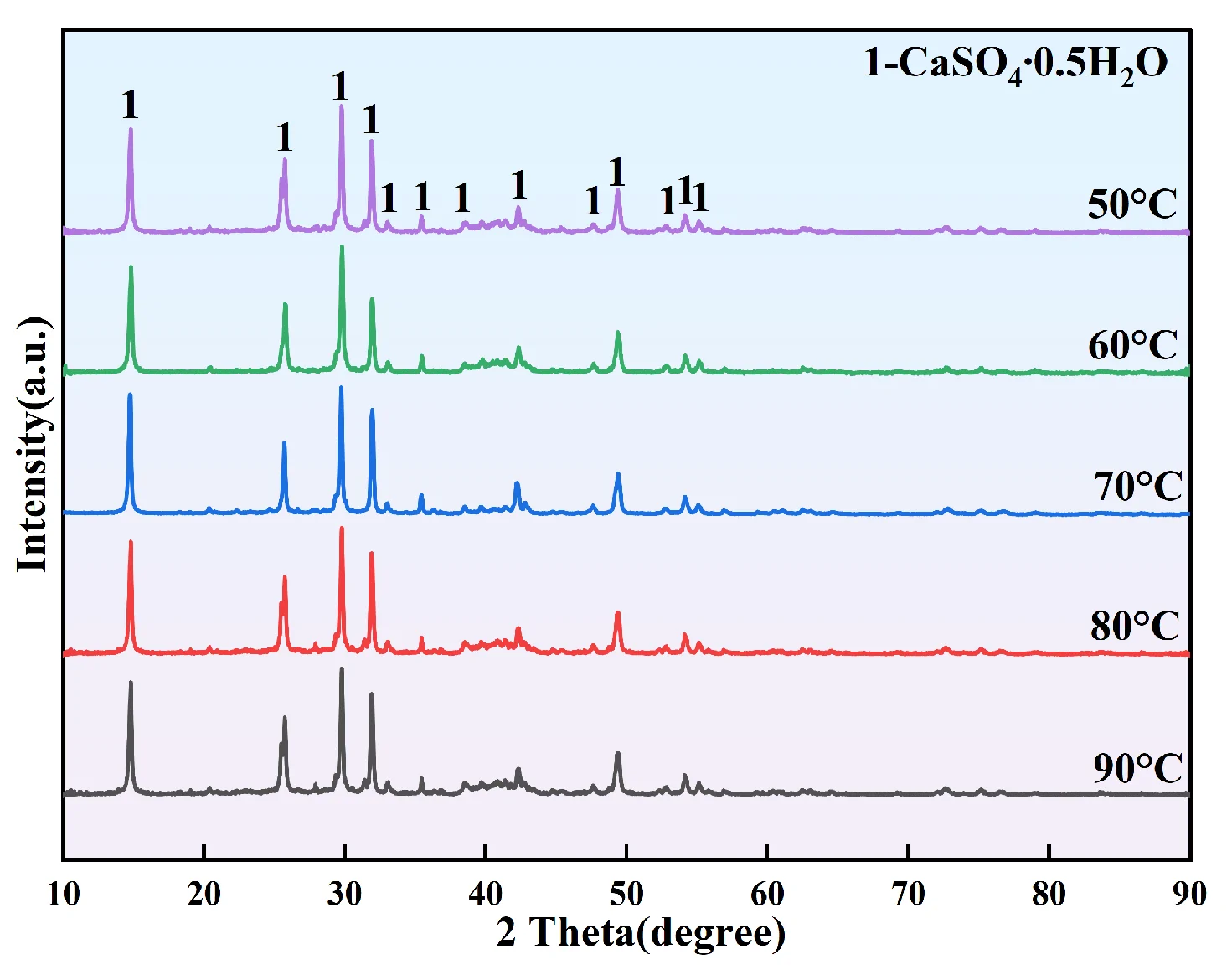
XRD patterns of residues at different leaching temperatures under microwave irradiation.

XRD analysis of residues under microwave-assisted conditions at different temperatures (Figure 6) indicates that all products are dominated by calcium sulfate hemihydrate. The intensity of the diffraction peak at 2θ ≈ 35.4° increases progressively with increasing temperature, suggesting improved crystallinity of the precipitated phase at elevated temperatures. Compared with the raw material, no obvious diffraction peaks attributable to C_2_F and FeO are observed, suggesting substantial decomposition of these original mineral phases and facilitating the transfer of vanadium into solution.

At lower temperatures, insufficient molecular kinetic energy limits vanadium release, whereas excessively high temperatures increase energy consumption and promote the dissolution of impurity ions, reducing leachate selectivity. Considering vanadium recovery rate, leachate selectivity, and process economy, 70 °C under microwave heating was identified as the optimal condition.

#### 2.2.3. Effect of Leaching Time on Vanadium Leaching

**Figure 7:**
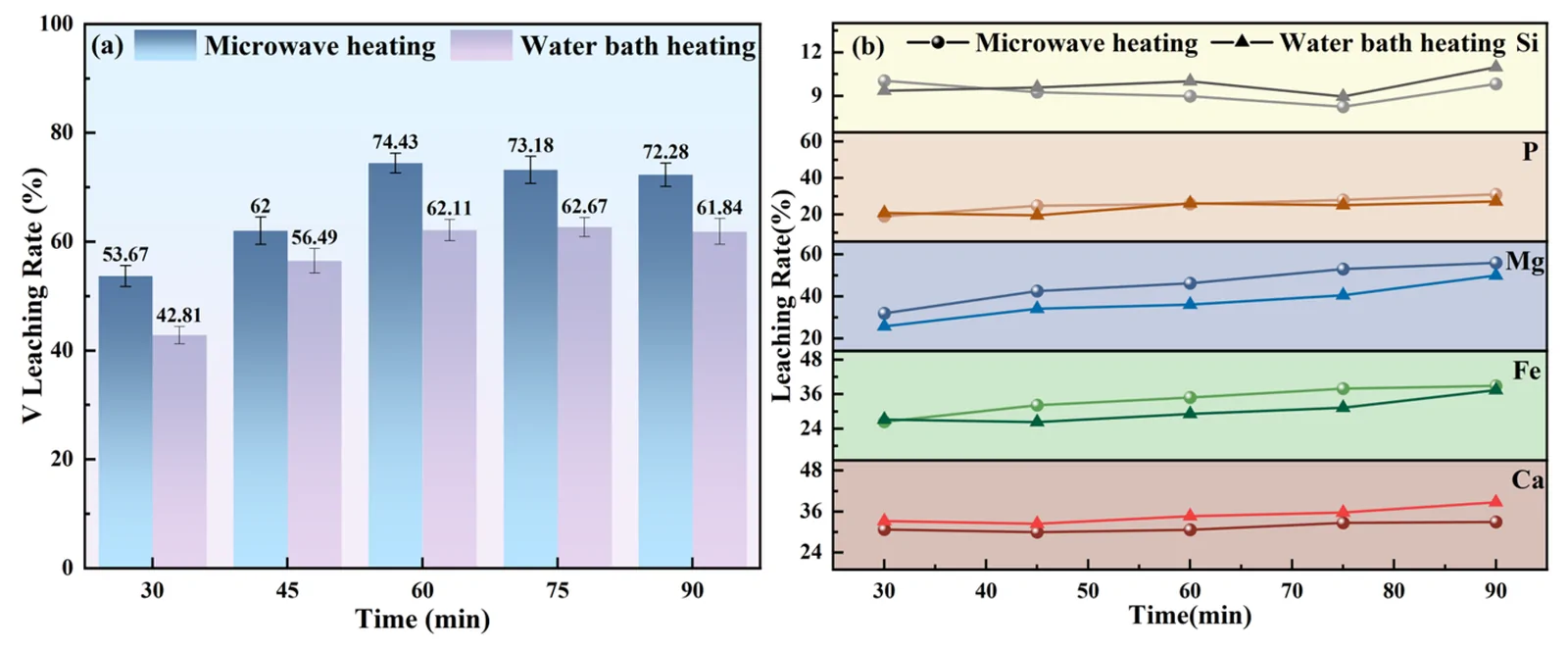
Effect of leaching time on the leaching rates of various elements under different heating methods: (**a**) vanadium leaching rate; (**b**) other elements leaching rates (sulfuric acid concentration: 15 wt.%, temperature: 70 °C, liquid-to-solid ratio: 8 mL·g^−1^).

Leaching time determines solid–liquid contact duration and is a key operating parameter governing the extent of acid attack on vanadium-bearing mineral phases, thereby affecting vanadium leaching behavior and overall leaching rate. Under fixed conditions of 15 wt.% sulfuric acid concentration, an L/S ratio of 8 mL·g^−1^, and a temperature of 70 °C, the influence of leaching time (30–90 min, at 15 min intervals) on vanadium leaching behavior was investigated, as shown in Figure 7.

As shown in Figure 7a, under both microwave-assisted and conventional water-bath heating, vanadium extraction rises swiftly as leaching time increases up to 60 min. This is attributed to the intensified solid–liquid interfacial reactions, where readily reactive vanadium-bearing phases on the surface of the pre-decalcified slag rapidly dissolve into solution.

When the leaching time reaches 60 min, the reaction between sulfuric acid and vanadium-bearing phases is essentially completed, and the vanadium concentration in solution approaches saturation, indicating that the system has reached a quasi-thermodynamic equilibrium state. At this point, the vanadium leaching rates under microwave-assisted and water-bath heating reach 74.43% and 62.11%, respectively. Further prolonging the leaching time does not significantly improve the vanadium leaching rate. Instead, it promotes the dissolution of impurity elements such as Mg and Fe (Figure 7b), resulting in diminished leachate selectivity and elevated downstream separation difficulty. Meanwhile, excessive reaction time reduces process throughput and increases operational cost, which is unfavorable for industrial application.

**Figure 8:**
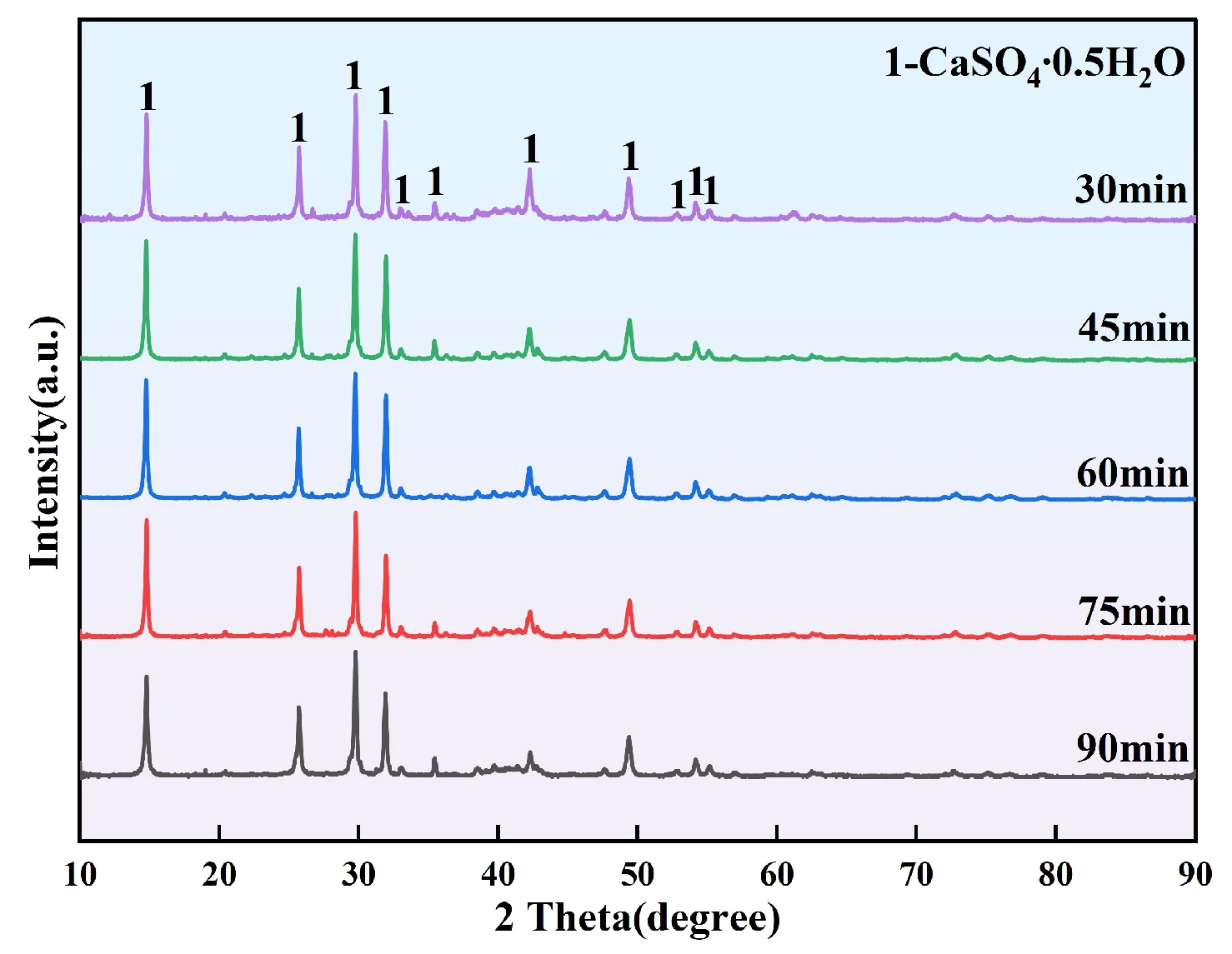
XRD patterns of residues at different leaching times under microwave irradiation.

XRD analysis of residues under microwave-assisted conditions at different leaching times (Figure 8) shows that all products are dominated by calcium sulfate hemihydrate. The relatively stable diffraction peak intensity indicates that the precipitation–dissolution equilibrium of Ca^2+^ and SO_4_^2−^ in solution is rapidly established during leaching. Vanadium is thus transferred into solution efficiently as the reactive phases are progressively decomposed.

At short leaching times, incomplete solid–liquid reactions lead to insufficient vanadium release, resulting in a lower leaching rate. In contrast, excessively long leaching times increase impurity dissolution and reduce leachate selectivity. Considering vanadium leaching rate, process efficiency, and impurity control, 60 min under microwave heating was identified as the optimal leaching time.

#### 2.2.4. Effect of Liquid-to-Solid Ratio on Vanadium Leaching

**Figure 9:**
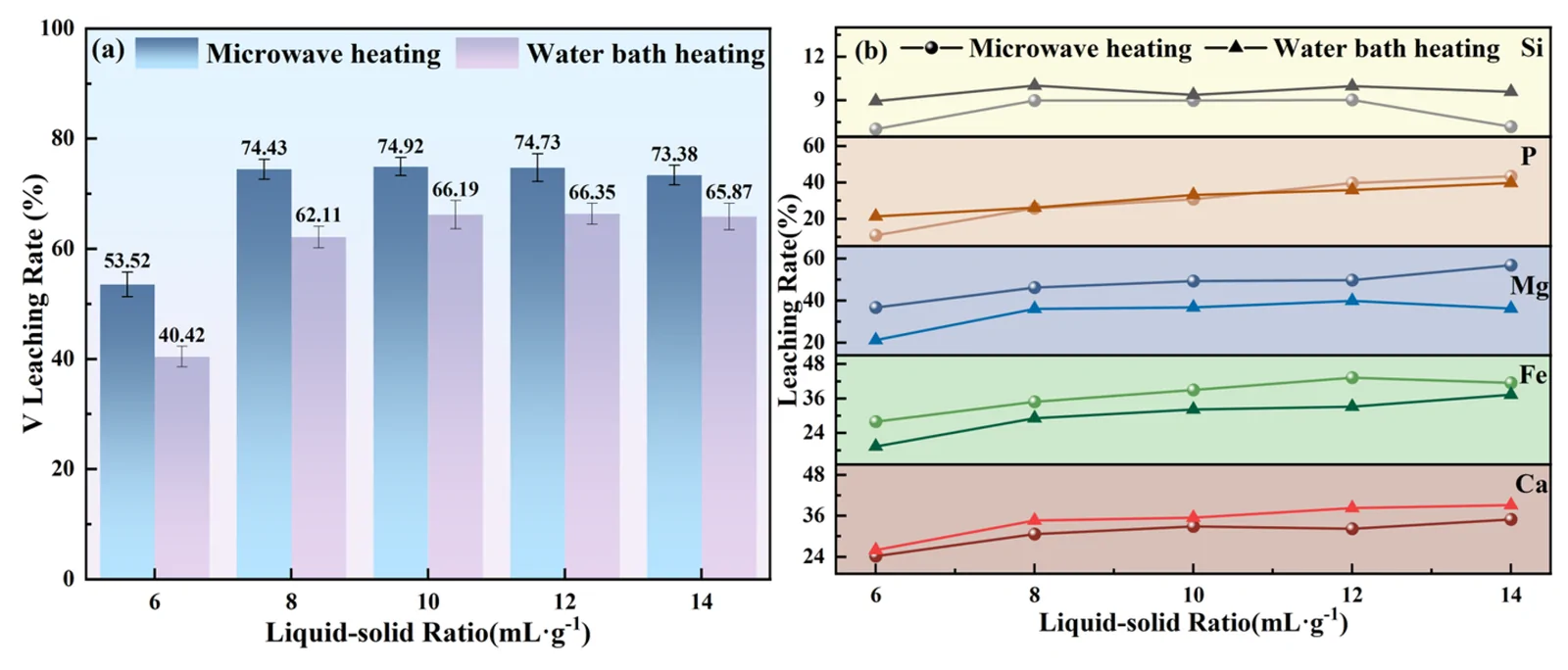
Effect of liquid-to-solid ratio on the leaching rates of various elements under different heating methods: (**a**) vanadium leaching rate; (**b**) other elements leaching rates (sulfuric acid concentration: 15 wt.%, temperature: 70 °C, time: 60 min).

The L/S ratio affects vanadium leaching behavior by influencing solution ion concentration and mass transfer efficiency. Under fixed conditions of 15 wt.% sulfuric acid concentration, 60 min, and 70 °C, the influence of L/S ratio (6–14 mL·g^−1^, interval of 2 mL·g^−1^) on vanadium leaching behavior was investigated, the results are presented in Figure 9.

As shown in Figure 9a, under both microwave-assisted and conventional water-bath heating, the vanadium leaching rate initially increases rapidly with increasing L/S ratio and then tends to plateau. At low L/S ratios, poor solid–liquid mixing and increased mass transfer resistance limit the effective contact between sulfuric acid and the pre-decalcified slag, resulting in a relatively low vanadium leaching rate. Under microwave-assisted conditions, as the L/S ratio increased from 6 to 8 mL·g^−1^, the improved dispersion of the solid phase and enhanced mass transfer promoted sufficient reaction between sulfuric acid and the slag matrix, leading to a rapid increase in the vanadium leaching rate from 53.52% to 74.43%. Further increasing the L/S ratio does not significantly improve vanadium leaching, indicating that the mass transfer conditions have reached an optimal state. At this stage, the L/S ratio is no longer the rate-limiting factor and a further increase only leads to unnecessary dilution of the leachate and reduced sulfuric acid utilization efficiency.

Under conventional water-bath heating, a similar trend is observed; however, the vanadium leaching rate is consistently lower than that under microwave irradiation across all conditions. This is attributed to the fact that water-bath heating relies mainly on macroscopic heat and mass transfer, making it more sensitive to diffusion limitations at low L/S ratios. In contrast, microwave irradiation promotes rapid volumetric heating and localized structural loosening, partially alleviating mass transfer limitations and thus enhancing vanadium dissolution efficiency.

As shown in Figure 9b, the leaching behaviors of impurity elements under both heating methods exhibit similar trends. The leaching of Si remains relatively stable with slight fluctuations, whereas Fe, Ca, Mg, and P show varying degrees of increase with increasing L/S ratio.

**Figure 10:**
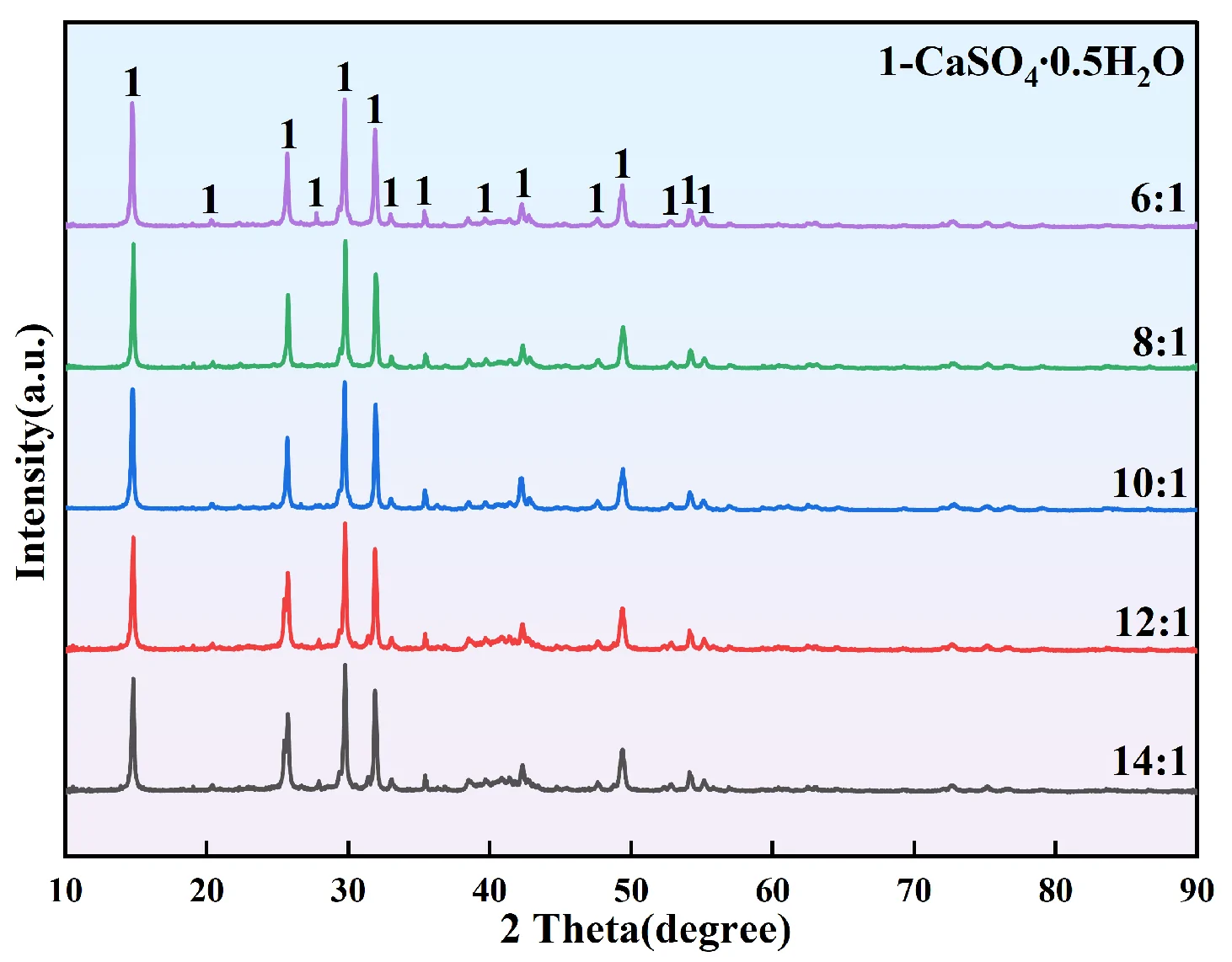
XRD patterns of residues at different liquid-to-solid ratios under microwave irradiation.

XRD analysis of residues under microwave-assisted conditions at different L/S ratios (Figure 10) shows that all leaching residues are composed exclusively of calcium sulfate hemihydrate. The diffraction peak intensity at 2θ ≈ 35.4° first increases and then stabilizes, reaching a maximum at an L/S ratio of 8 mL·g^−1^, indicating the establishment of a stable precipitation-dissolution equilibrium. No obvious diffraction peaks attributable to vanadium-bearing or Fe-rich phases are detected in the residues, suggesting substantial decomposition of these phases and the associated transfer of vanadium into solution.

At low L/S ratios, insufficient mass transfer leads to incomplete dissolution of vanadium-bearing phases, resulting in a lower leaching rate. At excessively high L/S ratios, although mass transfer is improved, sulfuric acid utilization efficiency decreases and impurity dissolution is promoted. Considering vanadium leaching rate, process economics, and impurity control, an L/S ratio of 8 mL·g^−1^ under microwave heating was identified as the optimum.

A comprehensive analysis of L/S ratio, leaching time, leaching temperature, and sulfuric acid concentration demonstrates that microwave-assisted leaching significantly enhances vanadium extraction under all investigated conditions compared with conventional water-bath heating, indicating a strong process intensification effect. Based on single-factor optimization, the optimum conditions for microwave-assisted leaching were established as 15 wt.% sulfuric acid, a temperature of 70 °C, a leaching time of 60 min, and an L/S ratio of 8 mL·g^−1^, under which a maximum vanadium leaching rate of 74.43% was achieved.

### 2.3. Countercurrent Leaching Process

**Figure 11:**
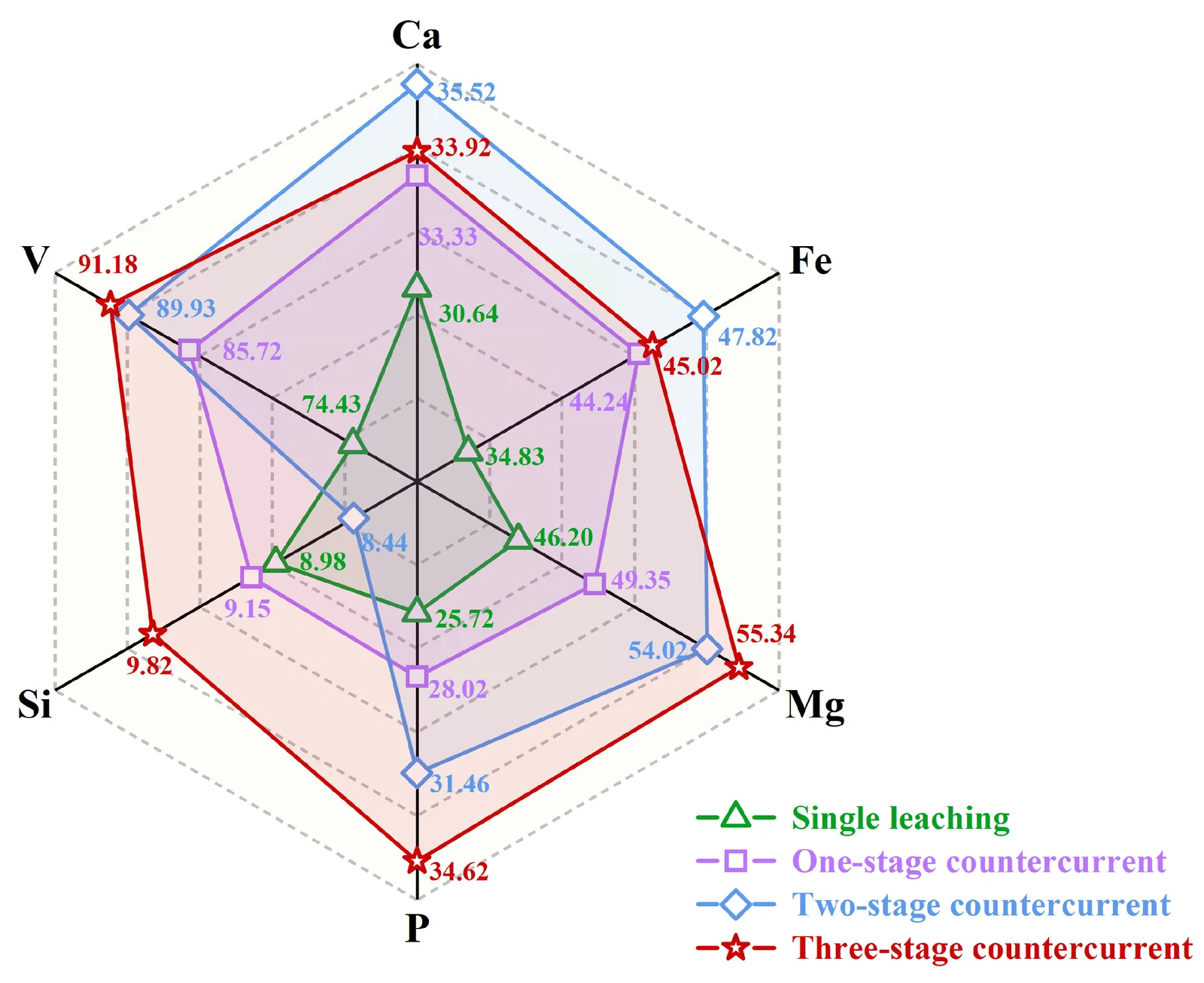
Cumulative leaching rates of various elements during the countercurrent leaching process (expressed as percentages).

To further enrich vanadium in the leachate, a batchwise multistage leachate-reuse procedure was employed to simulate countercurrent leaching. The leachate obtained from the initial leaching experiment under the optimal conditions was used as the leaching medium to treat a fresh batch of pre-decalcified slag at 70 °C for 60 min and an L/S ratio of 8 mL·g^−1^. After each leaching stage, the solid residue was separated, and the recovered leachate was directly reused to treat another fresh batch of pre-decalcified slag under the same conditions. The mass of fresh slag and the liquid volume were maintained constant at each stage, and the leachate-reuse procedure was continued until the vanadium concentration showed no appreciable further increase. The concentrations of V and impurity elements in the leachate after each stage were determined by ICP-OES. To quantify the accumulation of each element in the circulating leachate, a normalized enrichment index (*E_i_*_,*j*_) and an incremental enrichment (Δ*E_i_*_,*j*_) were defined, as shown in Equations (1) and (2):

(1) $$E_{i,j}=\frac{c_{i,j}\times V}{m\times w_{i}}\times 100\%,$$

(2) $$\Delta E_{i,j}=\frac{(c_{i,j}-c_{i,j-1})\times V}{m\times w_{i}}\times 100\%,$$

where *c_i_*_,*j*_ is the concentration of element *i* in the leachate after stage *j* (g·L^−1^), and *c_i_*_,0_ denotes the concentration of element *i* in the leachate obtained from the initial single-stage leaching experiment, *V* is the constant leachate volume (L), *m* is the mass of fresh pre-decalcified slag used at each stage (g), and *w_i_* is the mass fraction of element *i* in the fresh pre-decalcified slag (dimensionless). *E_i_*_,*j*_ represents the amount of element *i* accumulated in the circulating leachate relative to the total amount of the same element contained in one batch of fresh pre-decalcified slag (%), while Δ*E_i_*_,*j*_ represents the net incremental enrichment after stage *j*, expressed in percentage points. The corresponding results are shown in Figure 11.

Countercurrent leachate reuse progressively increased the accumulation of vanadium in the liquid phase. As shown in Figure 11, the normalized enrichment index of V increased from 74.43% after the initial single-stage leaching to 85.72% after the first reuse stage, corresponding to an incremental enrichment of 11.29 percentage points. After the second reuse stage, the vanadium concentration increased to 1.57 g·L^−1^ and the normalized enrichment index reached 89.93%, representing a further increase of 4.21 percentage points. In the third stage, the vanadium concentration increased only slightly to 1.59 g·L^−1^, while the normalized enrichment index reached 91.18%, corresponding to an additional increase of only 1.25 percentage points.

With increasing reuse stages, the normalized enrichment indices of P and Mg generally increased, indicating their progressive accumulation in the circulating leachate. In contrast, the Si enrichment index decreased slightly during the second stage, which may be associated with silica polymerization and re-adsorption of colloidal silica onto the solid surface. The Ca enrichment index decreased in the third stage, possibly because the increasing concentrations of Ca^2+^ and SO_4_^2−^ promoted secondary precipitation of calcium sulfate. A slight decrease in Fe was also observed in the third stage, which may be related to the progressive consumption of acidity and hydrolysis or precipitation of Fe-containing species during repeated heating.

Multistage leachate reuse progressively enriched vanadium in the circulating solution while further utilizing the residual leaching capacity of the solution. However, the incremental vanadium enrichment decreased markedly with increasing stage number. In particular, extending the process from the second to the third reuse stage increased the normalized V enrichment index only from 89.93% to 91.18%, while the vanadium concentration increased by only 0.02 g·L^−1^. Meanwhile, the accumulation of several impurity elements continued. Considering the diminishing vanadium enrichment, increasing impurity burden, and additional operating requirements associated with an extra leaching stage, the two-stage configuration was selected as the preferred operating condition.

### 2.4. Characterization of the Leaching Residue

The leaching residue obtained under the optimal conditions (leaching time of 60 min, L/S ratio of 8 mL·g^−1^, temperature of 70 °C, and sulfuric acid concentration of 15 wt.%) was analyzed by ICP-OES to determine the concentrations of the major elements, as summarized in Table 1, thereby providing a quantitative assessment of vanadium leaching selectivity and impurity co-leaching.

Under the optimal conditions, the leaching rate of V reached 74.43%, which was markedly higher than those of the major impurity elements. The corresponding leaching rates of Mg, Fe, Ca, P, and Si were 46.20%, 34.83%, 30.64%, 25.72%, and 8.98%, respectively. These results indicate that vanadium exhibited preferential dissolution relative to the major matrix and impurity elements. Nevertheless, the co-leaching of Mg and Fe remained appreciable, indicating that subsequent purification of the leachate is still necessary before vanadium recovery. In contrast, Si showed the lowest leaching rate, suggesting that most Si remained in the solid residue under the selected conditions.

**Figure 12:**
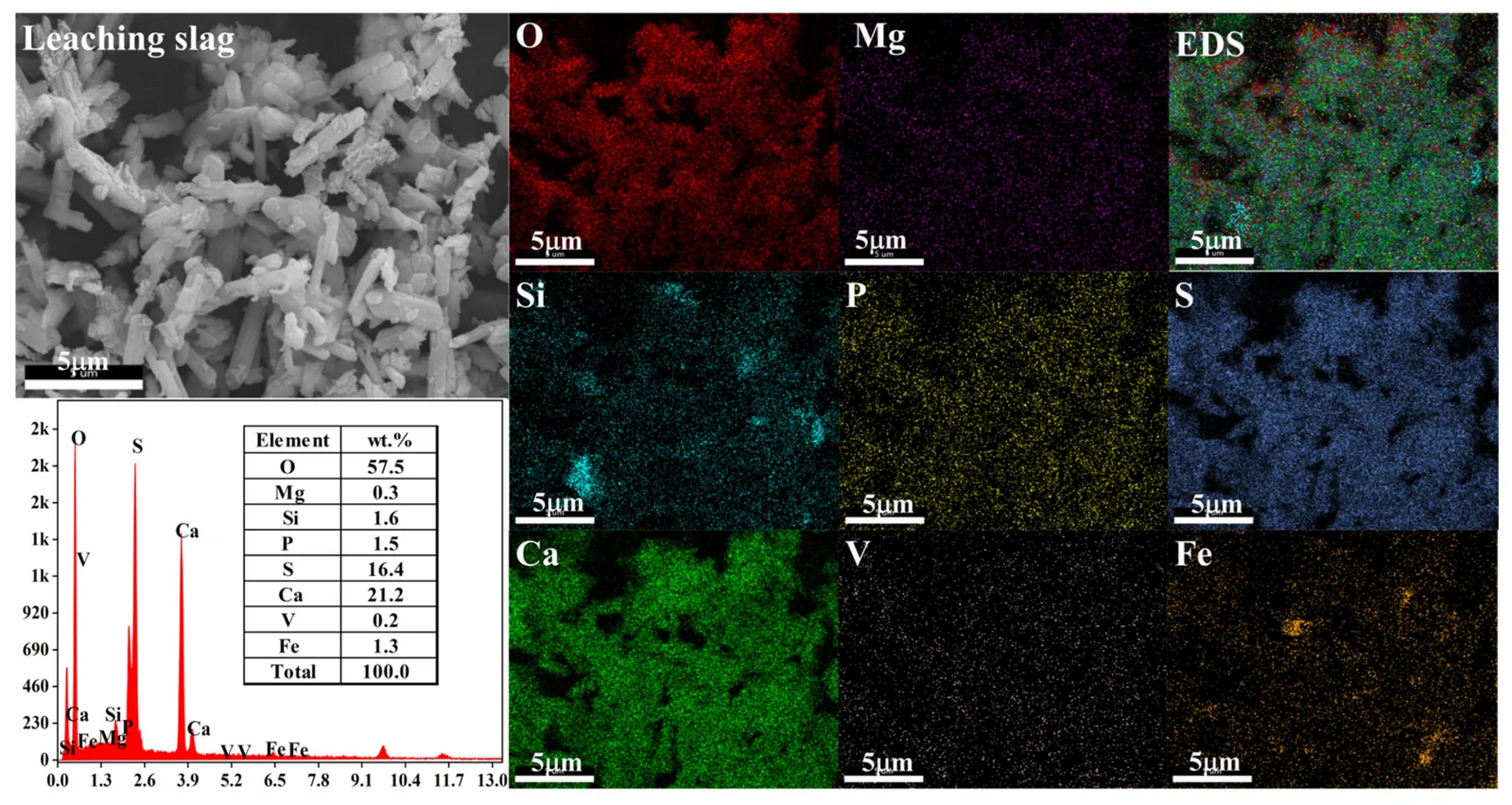
SEM image and EDS elemental mapping of the leaching residue obtained under optimal conditions.

**Figure 13:**
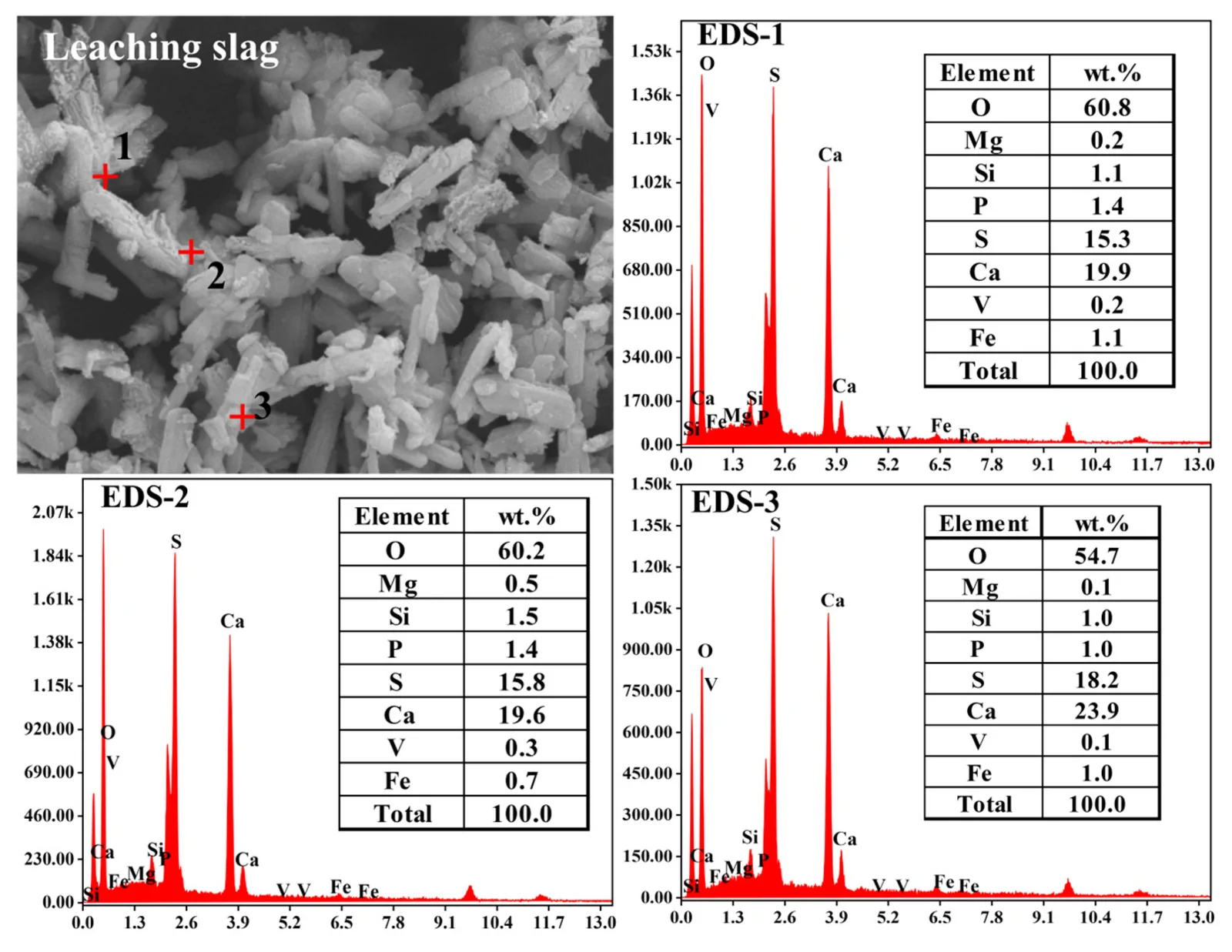
SEM image and EDS point analysis of the leaching residue.

In addition, the corresponding leaching residue was further characterized by SEM-EDS to investigate its microstructural morphology and elemental distribution, as shown in Figure 12 and Figure 13. The residue exhibits a dense, needle-like morphology with relatively smooth surfaces. In some regions, agglomerated structures are observed, which may encapsulate unreacted core particles and hinder further vanadium dissolution. Combined SEM-EDS mapping, point analysis, and XRD results confirm that the leached product is dominated by calcium sulfate phases. Trace elements like Fe, Mg, Si, and P are also detected within the matrix, while vanadium is highly dispersed and only present at trace levels, indicating that most vanadium has been effectively transferred into the leachate.

**Figure 14:**
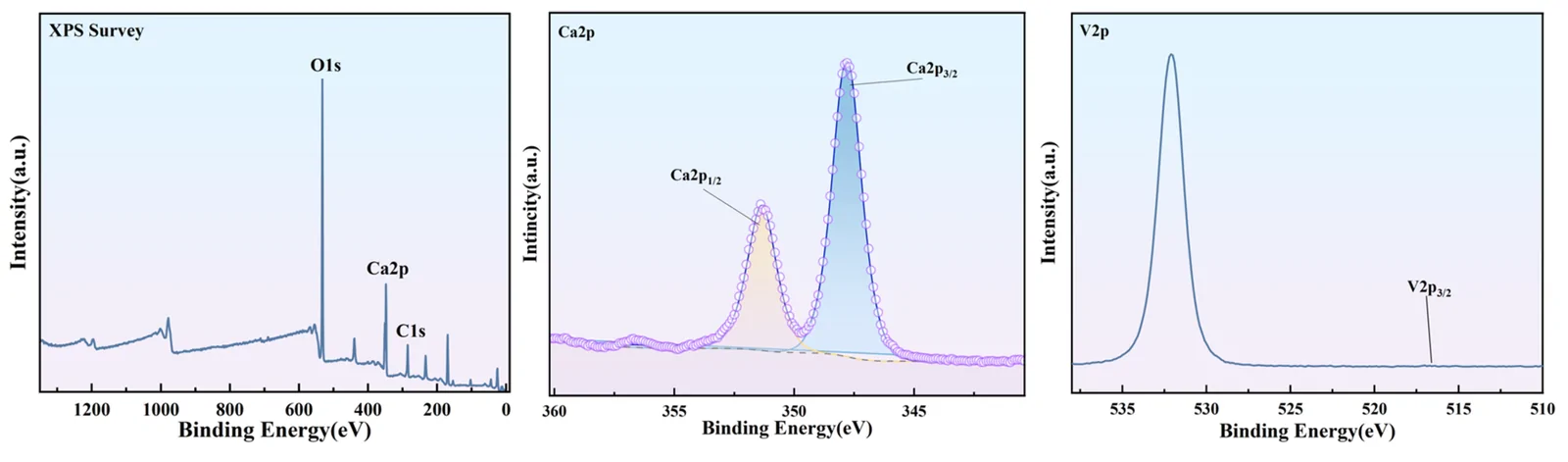
XPS spectra of the leaching residue.

XPS analysis further confirms the efficient removal of vanadium, as shown in Figure 14. The survey spectrum indicates a high Ca content and a very low V signal, demonstrating that vanadium is largely depleted from the solid phase. The Ca 2p spectrum exhibits a doublet at 347.8 eV (Ca2p_3/2_) and 351.3 eV (Ca2p_1/2_), attributable to Ca^2+^ in CaSO_4_ [20], confirming that calcium is immobilized in the residue as sulfate species after leaching.

The V 2p spectrum exhibits only a very weak signal, with the V 2 p_3/2_ peak located at 516.4 eV. Due to the extremely low concentration approaching the detection limit, no peak deconvolution was performed. The significant attenuation of the V signal directly confirms the effective extraction of vanadium during the acid leaching process.

From a mechanistic perspective, sulfuric acid provides H^+^ ions that attack the residual calcium-bearing silicate phases in the pre-decalcified slag, breaking Ca-O-Si bonds and destabilizing the crystal lattice. This structural collapse releases V^5+^, which substitutes for Si sites or resides at phase boundaries in tetrahedral coordination environments. Simultaneously, H^+^ also reacts with calcium ferrite phases, breaking Ca-O-Fe bonds and exposing V^4+^ species originally occupying octahedral Fe sites.

Due to the different acid solubilities of dicalcium silicate and calcium ferrite, the dissolution of the former is faster, resulting in different release kinetics of vanadium from distinct host phases. Vanadium released from the solid phase may exist in different oxidation states. Under acidic and sufficiently oxidizing conditions, V^4+^ may be further oxidized to V^5+^, with V^4+^ and V^5+^ represented mainly by VO^2+^ and VO_2_^+^, respectively, in solution.

Beyond the leaching performance and residue characteristics discussed above, several practical considerations should also be addressed for the potential application of the proposed process. Although the proposed pre-decalcification–microwave-assisted leaching process shows promising vanadium recovery performance, several practical limitations should be considered for further application. Pre-decalcification removes a substantial fraction of Ca-containing phases before sulfuric acid leaching, which can reduce the consumption of sulfuric acid by acid-consuming matrix components and is also beneficial for subsequent vanadium extraction. Nevertheless, sulfuric acid consumption remains an important factor affecting reagent cost and downstream solution treatment. The leaching residue is mainly enriched in Ca- and S-bearing phases, particularly calcium sulfate, suggesting potential utilization as a raw material for cementitious or other construction materials [21]. However, further evaluation of its chemical stability, residual metal contents, and environmental safety is required before practical utilization. In addition, corrosion of equipment exposed to hot sulfuric acid solutions should be considered when selecting reactor materials. For scale-up of microwave-assisted leaching, microwave penetration depth, non-uniform electromagnetic-field distribution, local overheating, and reactor geometry may affect heating uniformity and energy efficiency [22]. Therefore, further optimization of reactor design and systematic evaluation of microwave energy consumption are necessary before industrial application.

### 2.5. Kinetic Analysis

To elucidate the reaction kinetics of vanadium leaching from pre-decalcified slag under microwave-assisted sulfuric acid leaching, the shrinking-core model assuming invariant particle dimensions [23] was employed to investigate how sulfuric acid concentration and reaction temperature affect the leaching kinetics.

For heterogeneous liquid-solid reactions, three kinetic models were considered, including diffusion control through the solid product layer, chemically controlled surface reactions, and mixed control, for discerning the rate-limiting mechanism:

For internal diffusion control:

1 − (2/3)*x* − (1 − *x*)^2/3^ = *k_d_t*, (3)

For surface chemical reaction control:

1 − (1 − *x*)^1/3^=*k_d_t*, (4)

For mixed control:

(1 − *x*)^−1/3^ − 1 + 1/3ln(1 − *x*)=*k_d_t*, (5)

where *x* is the vanadium leaching rate (%), *t* is the reaction time (min), and *k_d_* is the apparent rate constant corresponding to each controlling step (min^−1^).

The temperature dependence of the apparent rate constant (*k_d_*) follows the Arrhenius equation:

(6) $$k_{d}=A\exp\left(-\frac{E_{a}}{RT}\right),$$

Taking the natural logarithm of both sides yields:

(7) $$\ln K_{d}=-\frac{E_{a}}{RT}+\ln A,$$

where *E_a_* is the apparent activation energy (kJ·mol^−1^), generally considered to be independent of temperature, R is the universal gas constant (8.314 J·mol^−1^·K^−1^), *T* is the absolute temperature (K), A is the pre-exponential factor (s^−1^).

#### 2.5.1. Effect of Sulfuric Acid Concentration on Vanadium Leaching Rate

**Figure 15:**
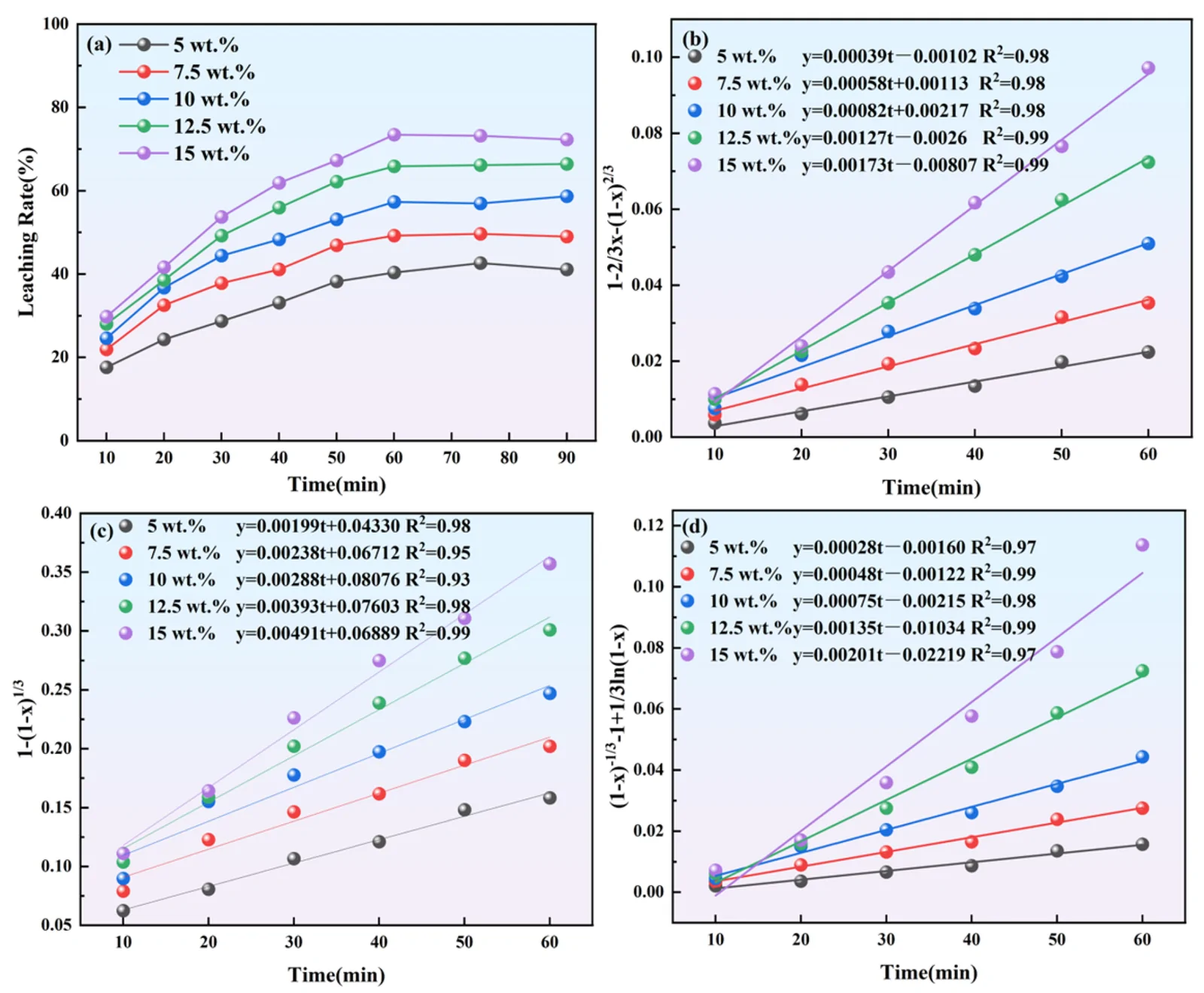
Kinetic fitting curves of the vanadium leaching process at different sulfuric acid concentrations: (**a**) effect of sulfuric acid concentration on vanadium leaching; (**b**) linear relationships between 1 − 2/3*x* − (1 − *x*)^2/3^ and *t*; (**c**) linear relationships between 1 − (1 − x)^1/3^ and *t*; (**d**) linear relationships between (1 − *x*)^−1/3^ – 1 + 1/3ln(1 − *x*) and *t* (temperature: 70 °C, time: 60 min, liquid-to-solid ratio: 8 mL·g^−1^).

Under fixed conditions of an L/S ratio of 8 mL·g^−1^, 60 min, and70 °C, the impact of sulfuric acid concentration (5–15 wt.%, in 2.5 wt.% increments) on the kinetic behavior of vanadium leaching was investigated. Figure 15a displays how vanadium extraction varies with acid concentration. During the initial 0–60 min stage, the vanadium leaching rate increased markedly with increasing sulfuric acid concentration, following an approximately linear dependence on acid concentration. Beyond 60 min, the leaching rate gradually reached a plateau, and no further significant variation was observed, indicating that the system had approached saturation under the given conditions. Therefore, only the data obtained within the first 60 min were used for kinetic fitting to identify the rate-limiting step.

As illustrated in Figure 15b–d, regression results reveal that both the internal diffusion control model (Figure 15b) and the mixed control model (Figure 15d) exhibit strong linear correlations (R^2^ > 0.95). A comparison of fitting quality shows that the internal diffusion model outperforms the mixed control model in terms of linear fitting. Although the chemical reaction control model (Figure 15c) also yields a correlation coefficient above 0.90, its linearity is inferior to that of the other two models, indicating weaker fitting performance under the present conditions. Based on these results, the leaching process within the investigated sulfuric acid concentration range is well represented by a size-invariant shrinking-core model, in which internal diffusion constitutes the primary rate-determining step.

**Figure 16:**
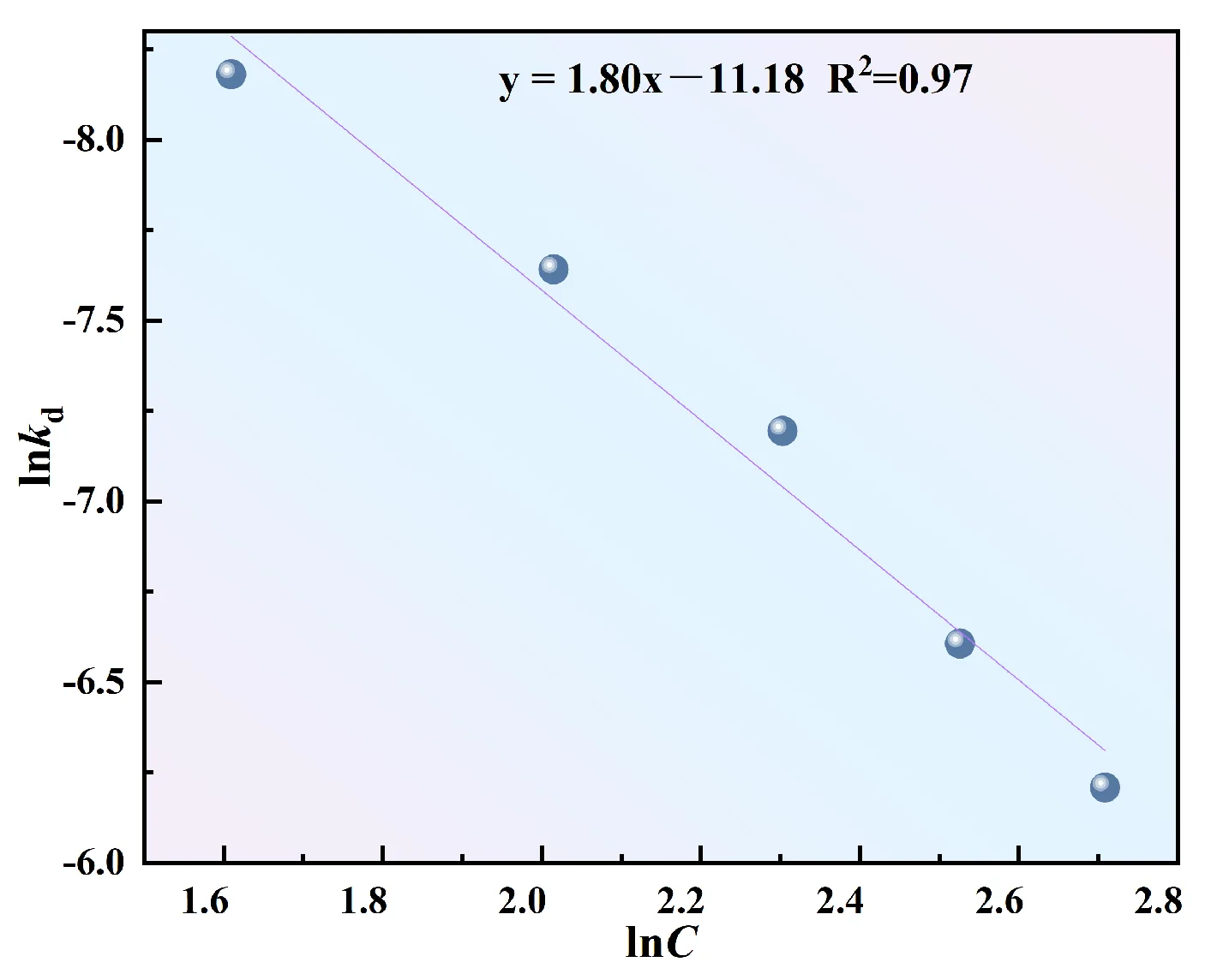
Relationship between the apparent rate constant and the logarithm of sulfuric acid concentration.

To further clarify the effect of sulfuric acid concentration on the leaching rate, the apparent rate constants *k*_d_ were calculated from the slopes of the fitted equations in Figure 15b under different conditions. The relationship between ln*k*_d_ and lnC is shown in Figure 16. the apparent reaction order was 1.80, and the semi-empirical kinetic expression was established as ln*k*_d_ = 1.80lnC − 11.18.

#### 2.5.2. Effect of Leaching Temperature on Vanadium Leaching Rate

**Figure 17:**
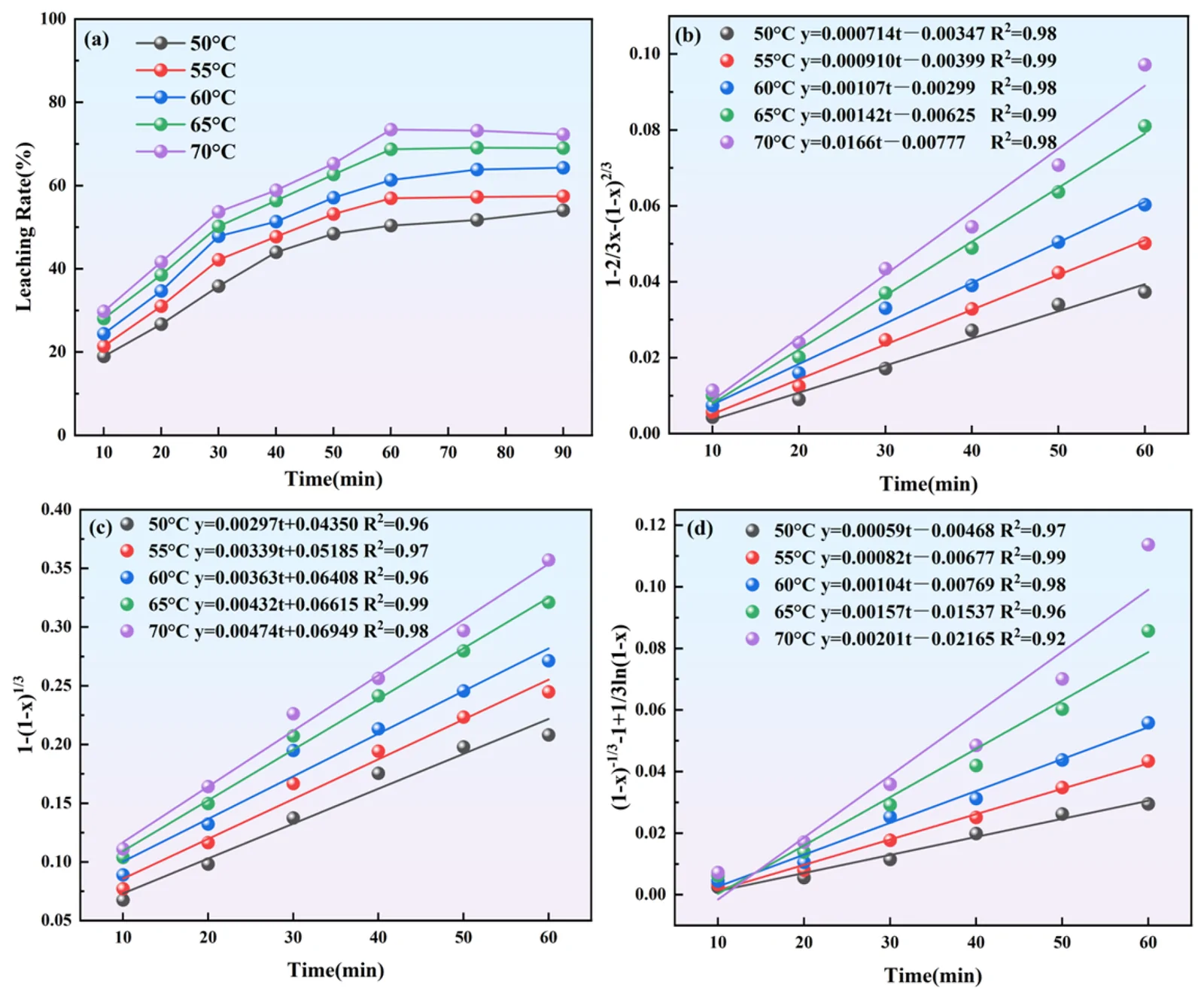
Kinetic fitting curves of the vanadium leaching process at different temperatures: (**a**) effect of leaching temperature on vanadium leaching; (**b**) linear relationships between 1 − 2/3*x* − (1 − *x*)^2/3^ and *t*; (**c**) linear relationships between 1 − (1 − x)^1/3^ and *t*; (**d**) linear relationships between (1 − *x*)^−1/3^ – 1 + 1/3ln(1 − *x*) and *t* (sulfuric acid concentration: 15 wt.%, time: 60 min, liquid-to-solid ratio: 8 mL·g^−1^).

Under fixed conditions of a leaching time of 60 min, 15 wt.% sulfuric acid concentration, and an L/S ratio of 8 mL·g^−1^, the effect of leaching temperature (50–70 °C, at 5 °C intervals) on the kinetic behavior of vanadium leaching was investigated. Figure 17a presents the temperature dependence of vanadium leaching.

During the initial 0–60 min stage, the vanadium leaching rate increased markedly with increasing temperature, showing an approximately linear dependence on temperature. Beyond 60 min, the leaching rate gradually reached a plateau and no further significant variation was observed, indicating that the system had approached saturation under the given conditions. Therefore, only the data within the first 60 min were selected for kinetic analysis, as illustrated in Figure 17b–d, to determine the rate-controlling step.

As shown in Figure 17b–d, both the internal diffusion control model (Figure 17b) and the chemical reaction control model (Figure 17c) exhibit strong correlations. However, the internal diffusion model shows a higher R^2^ value than the chemical reaction model, indicating a better fitting performance. Although the mixed control model (Figure 17d) also yields correlation coefficients above 0.90, its overall linearity is relatively weaker.

These results suggest that, within the investigated thermal window (50–70 °C), leaching behavior follows a constant-particle-size shrinking-core model, predominantly governed by internal diffusion.

**Figure 18:**
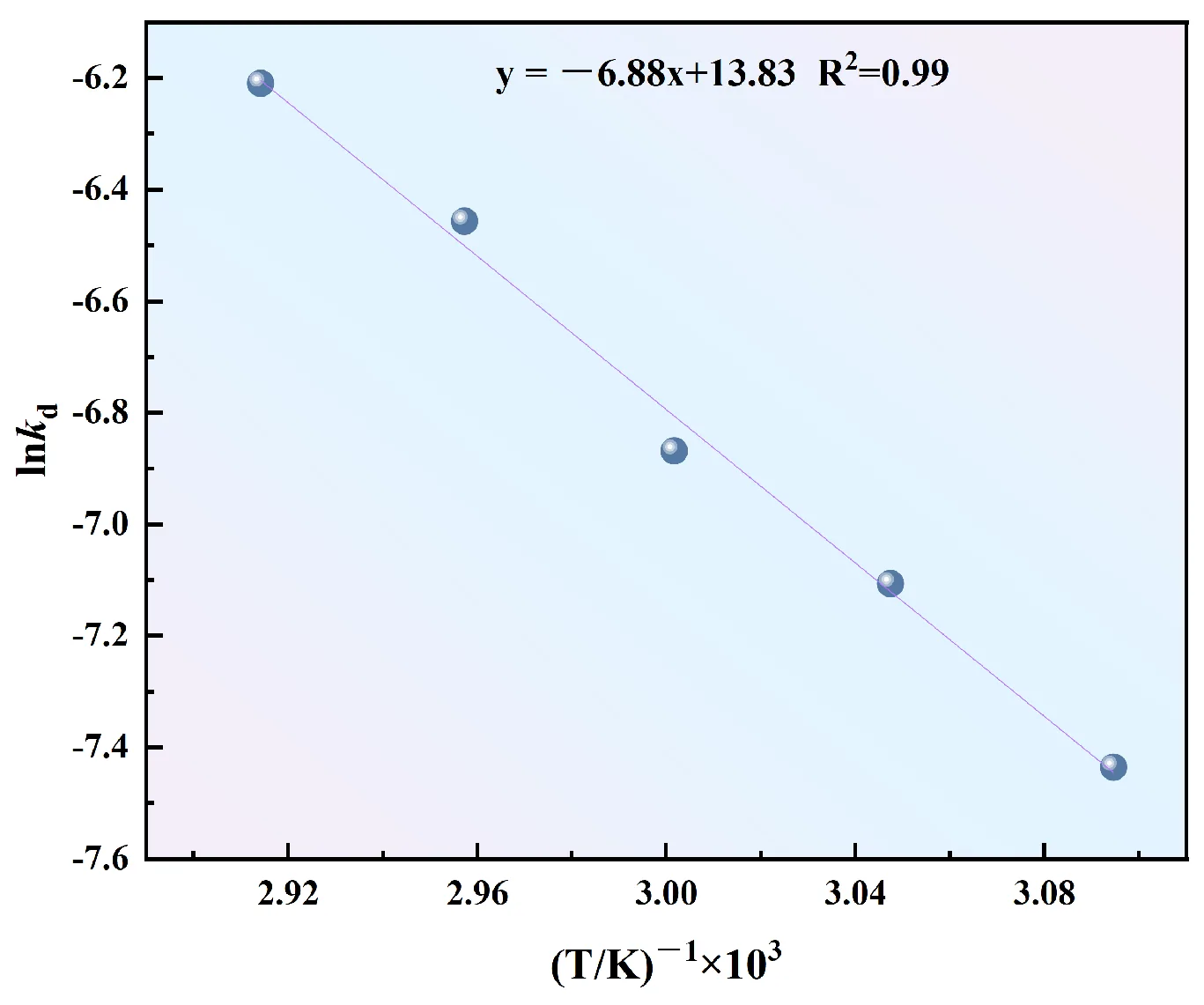
Arrhenius plot of ln *k*_d_ versus 1000/*T* for the vanadium leaching process.

To further assess this kinetic interpretation, *E*_a_ was determined from the Arrhenius treatment of the temperature-dependent rate constants, as shown in Figure 18. The apparent activation energy for microwave-assisted sulfuric acid leaching was calculated to be 57.20 kJ·mol^−1^. The apparent activation energy can be used as an auxiliary criterion for identifying the rate-controlling mechanism. Generally, diffusion-controlled, mixed-controlled, and chemical-reaction-controlled processes are associated with apparent activation energies of approximately 4–12, 12–40, and 40–300 kJ·mol^−1^, respectively [24]. The *E*_a_ obtained in the present study is therefore higher than the ranges generally associated with diffusion- and mixed-controlled processes, indicating that the leaching process cannot be described solely by product-layer diffusion. Nevertheless, among the three kinetic models examined, the internal-diffusion model exhibited the highest regression coefficients, indicating that internal diffusion remained the predominant rate-controlling step. Therefore, the leaching process is more reasonably interpreted as being predominantly controlled by internal diffusion, while the contribution of the interfacial chemical reaction cannot be neglected.

Similar coupled kinetic behavior has also been reported for vanadium-bearing steel slag. Liu et al. investigated pressure acid leaching of roasted vanadium-bearing steel slag and reported an apparent activation energy of 20.87 kJ·mol^−1^. Their results indicated that both interfacial transfer and diffusion through the product layer affected the vanadium leaching rate [25]. Compared with that study, the apparent activation energy obtained in the present work is higher, indicating that the present system exhibits stronger temperature dependence. According to the kinetic-model fitting results, internal diffusion is the predominant rate-controlling step, while the interfacial chemical reaction also makes a non-negligible contribution.

## 3. Materials and Methods

### 3.1. Materials

The vanadium-bearing steel slag used in this study was obtained from a steel plant in Chengde, Hebei Province, China. After grinding, the raw slag exhibited a D10–D90 particle-size range of 10.542–490.765 μm, with a median particle size (D50) of 146.870 μm. The pre-decalcified slag obtained under the optimal HCl pretreatment conditions (1.5 mol·L^−1^ HCl, 40 °C, 8 min, and a liquid-to-solid ratio of 7 mL·g^−1^) served as the feedstock for microwave-assisted leaching. Under these conditions, the Ca removal rate reached 77.10%. The pre-decalcified slag exhibited a D10–D90 particle-size range of 10.163–452.151 μm, with a median particle size (D50) of 110.167 μm. After digestion, the chemical composition of the pre-decalcified slag was quantitatively analyzed using inductively coupled plasma optical emission spectroscopy (ICP-OES, 725 ES, Agilent Technologies, Mulgrave, VIC, Australia). The chemical compositions of the raw and pre-decalcified slags are compared in Table 2. Compared with the raw slag, the CaO content decreased markedly after pre-decalcification, while V was largely retained in the solid phase. In the pre-decalcified slag, Fe_2_O_3_ was the dominant component, accounting for 34.45 wt.%, while the target component V_2_O_5_ was 2.49 wt.%.

**Figure 19:**
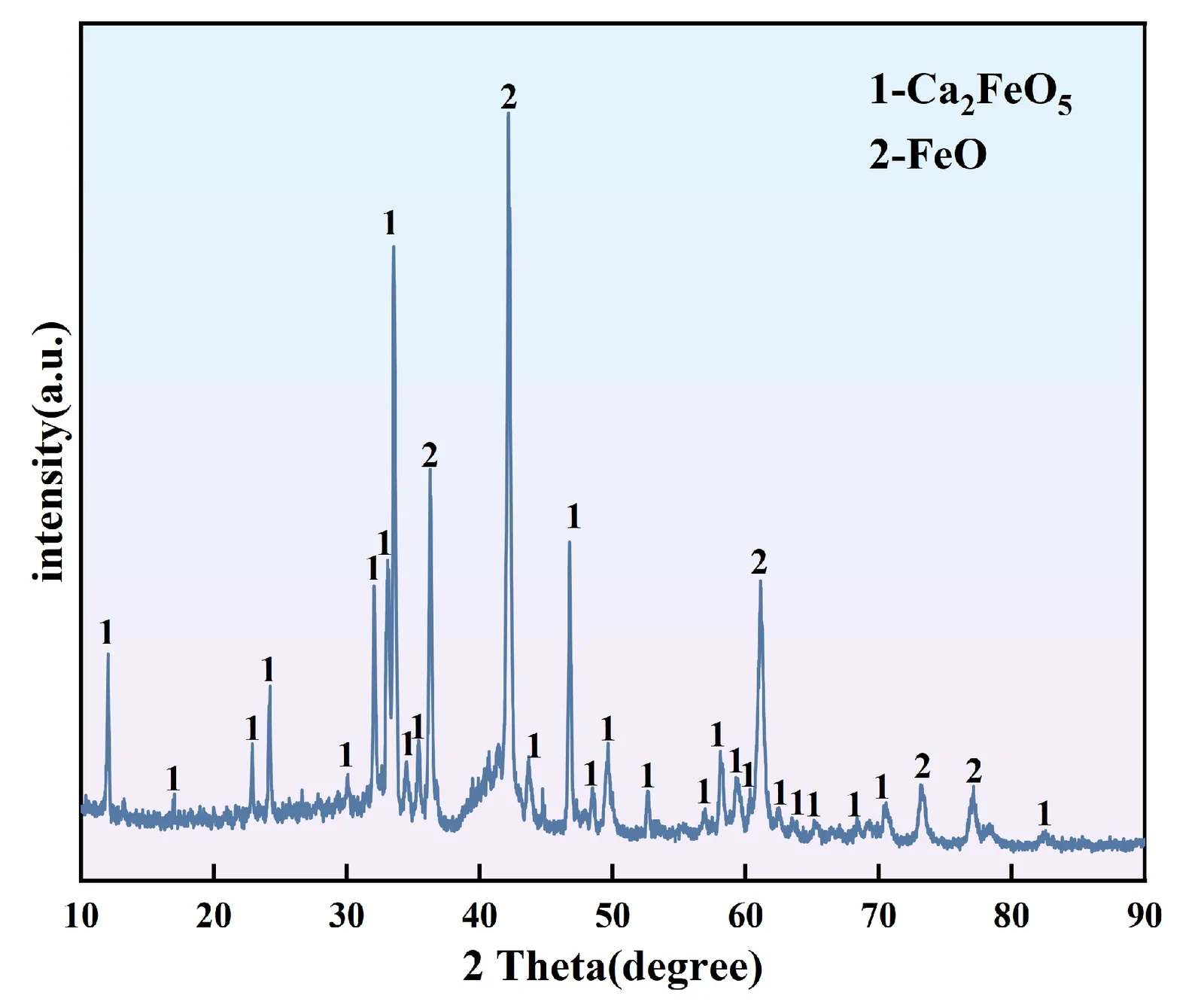
XRD pattern of the pre-decalcified vanadium-bearing steel slag.

The mineralogical composition of the pre-decalcified slag was characterized using X-ray diffraction (XRD, D/MAX2500PC, Rigaku, Akishima, Japan), and the results are presented in Figure 19. The results indicate that the pre-decalcified slag is mainly composed of dicalcium ferrite (C_2_F) and FeO, while no independent crystalline vanadium oxide phase was detected.

**Figure 20:**
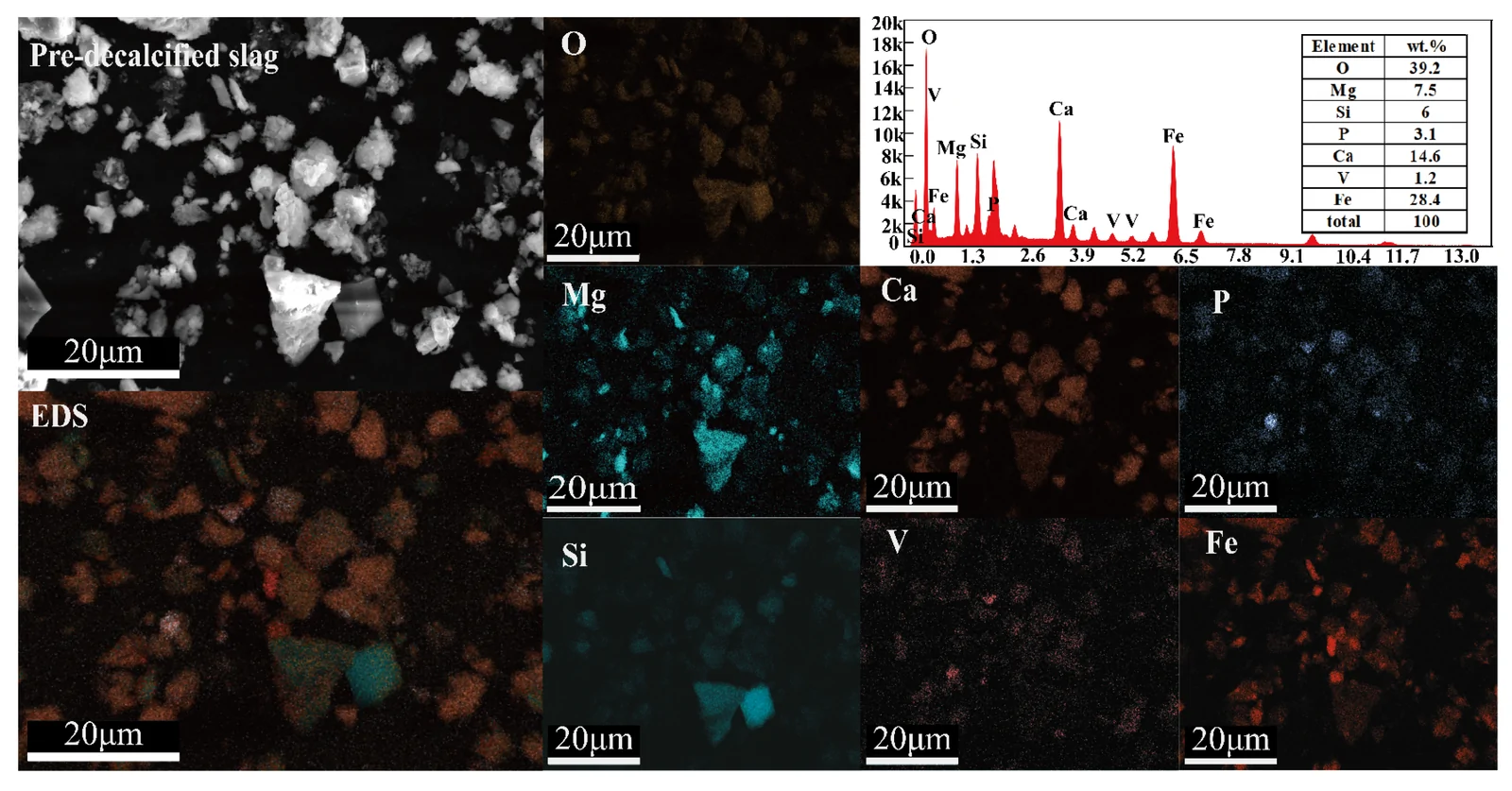
SEM image and elemental mapping of the pre-decalcified slag.

**Figure 21:**
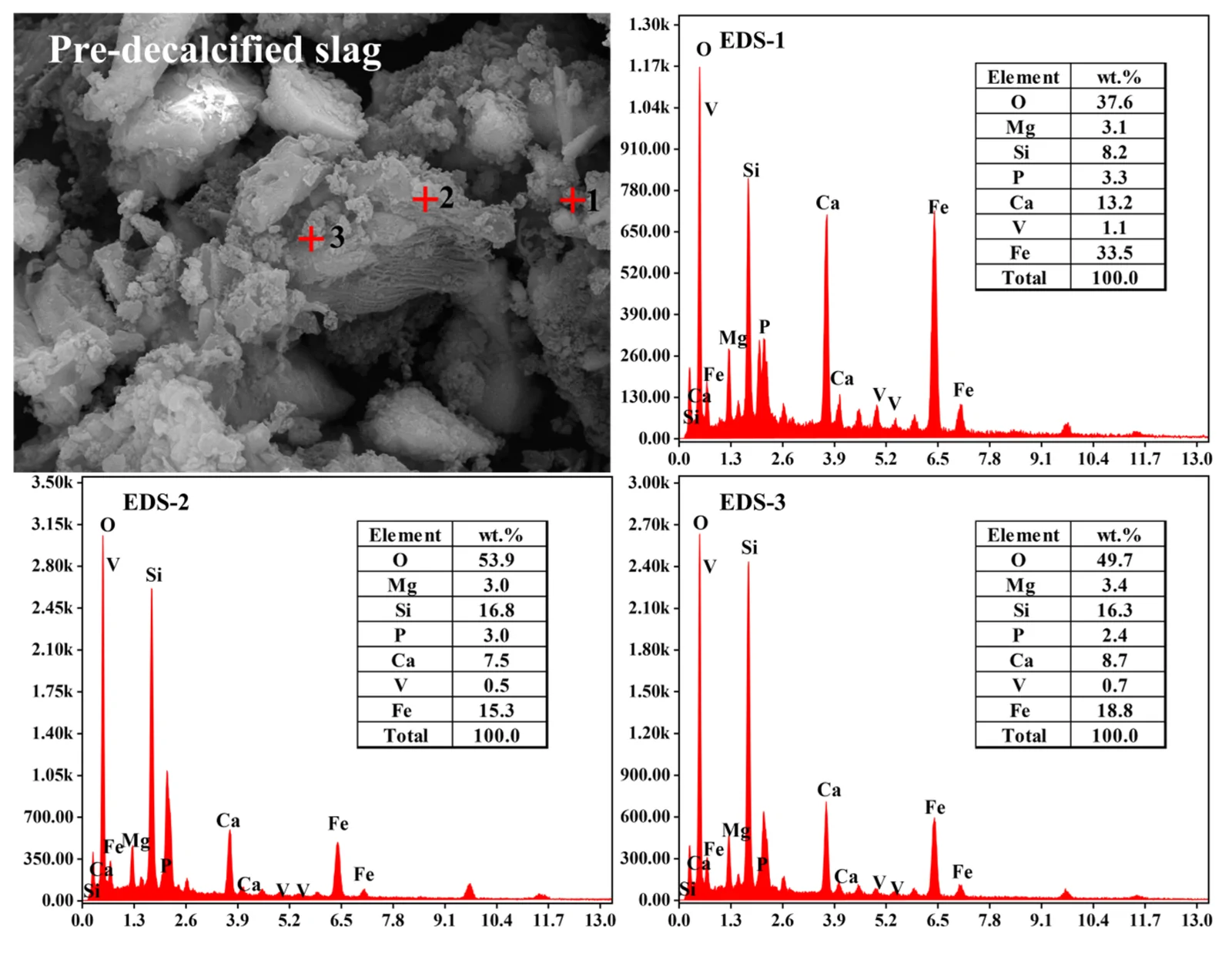
SEM image and EDS point analysis of the pre-decalcified slag.

To further determine the occurrence state of vanadium, scanning electron microscopy coupled with energy-dispersive spectroscopy (SEM-EDS, Quattro S, Thermo Fisher Scientific, Hillsboro, OR, USA; EDAX Element Plus, AMETEK, Mahwah, NJ, USA) was employed to analyze the microstructure and elemental composition of the pre-decalcified slag. The SEM images and EDS mapping results are shown in Figure 20. The pre-decalcified slag exhibited a relatively loose and porous morphology, with obvious local corrosion features observed on some particle surfaces, indicating the preferential dissolution of Ca-bearing phases during HCl pretreatment. The elemental mapping results showed obvious local enrichment of Fe. Ca, Fe, and O exhibited a relatively high degree of spatial overlap in some regions, which, combined with the XRD results, is inferred to correspond mainly to calcium ferrite phases. In addition, regions with pronounced overlap between Fe and O were observed, which, together with the XRD results, are inferred to be associated with FeO phases. In the lower-central region of the mapping area, Si and O exhibited strong spatial overlap, while the Ca signal was relatively weak, suggesting the presence of a Si-rich residual framework after decalcification. V shows a dispersed distribution overall. Point analysis (Figure 21) combined with XRD results indicates that the selected regions are mainly composed of Fe and Si with minor Ca content, suggesting their association with Fe-rich phases (e.g., calcium ferrite and ferrous oxide) and calcium-deficient silicate skeletons.

**Figure 22:**
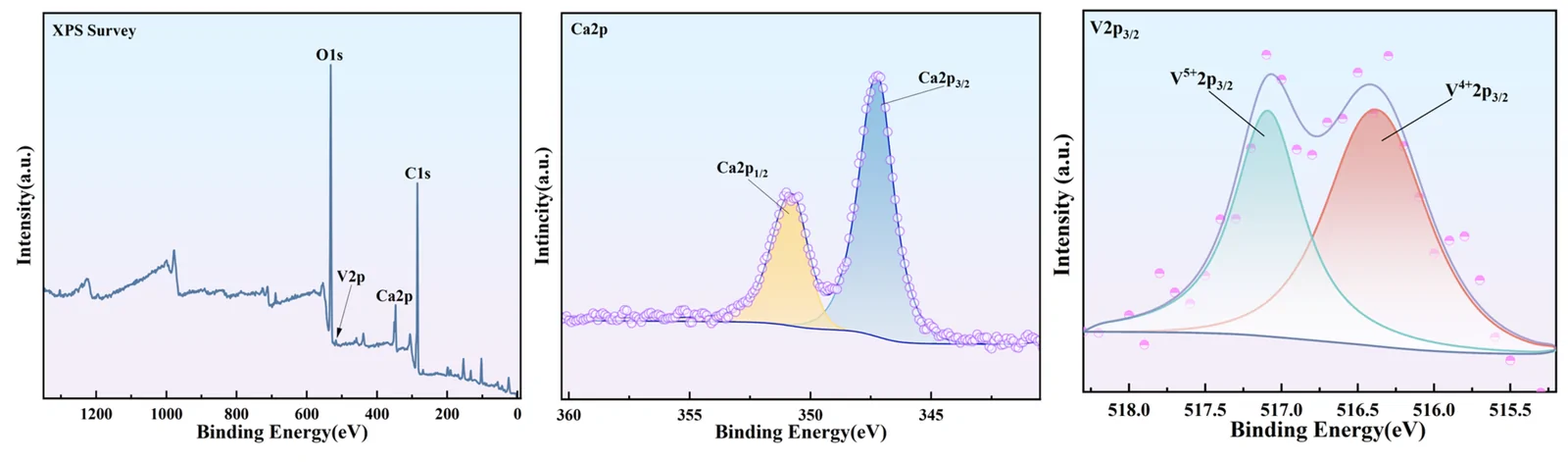
XPS spectra of the pre-decalcified slag.

The chemical states of Ca and V in the pre-decalcified slag were investigated by X-ray photoelectron spectroscopy (XPS, K-Alpha, Thermo Fisher Scientific, USA), with the resulting spectra shown in Figure 22. The survey spectrum indicates that the slag surface is mainly composed of C, O, Ca, and V. The C 1s peak at 284.8 eV originates from adventitious carbon and was used for charge correction.

The Ca 2p spectrum exhibits a typical spin–orbit doublet with components at 347.2 eV (Ca 2p_3/2_) and 350.7 eV (Ca 2p_1/2_), indicating that Ca exists in the form of Ca^2+^ within silicate and ferrite lattices [26], corresponding to Ca-O bonding environments in C_2_S and C_2_F phases.

The pre-decalcified slag contains vanadium predominantly as V^5+^ and V^4+^ species, which are incorporated into C_2_S and C_2_F phases via isomorphous substitution. V^5+^ is suggested to be partially associated with tetrahedral Si sites and enriched near the C_2_S boundary regions, exhibiting a relatively higher oxidation state. V^4+^ is considered to substitute for Fe^3+^ in octahedral coordination [27]. The V 2p spectrum shows a V 2p_3/2_ peak centered at 517.1 eV, assignable to V^5+^ [28]. Because of low vanadium content, the V 2p_1/2_ signal is weak and cannot be clearly resolved; therefore, only the V 2p_3/2_ peak was used for fitting. Furthermore, a weak peak at 516.4 eV was resolved, attributed to V^4+^ species, likely originating from octahedral substitution of Fe^3+^ in the C_2_F phase.

### 3.2. Experimental Methods

(1) Leaching of vanadium from pre-decalcified slag

A 5 g portion of the pre-decalcified slag was mixed with sulfuric acid solutions of different concentrations (5–25 wt.%) at L/S ratios of 6–14 mL·g^−1^ in a round-bottom flask. The flask was then placed in a microwave reactor (WBMW-H2, Tangshan Renshi Juyuan Microwave Apparatus Co., Ltd., Tangshan, China) operating at a microwave frequency of 2.45 GHz ± 50 MHz with a rated power of 2 kW. The experiments were conducted under microwave-assisted constant-temperature heating with continuous stirring at 400 r·min^−1^. The solution temperature was measured at the center of the liquid phase using a temperature sensor specifically designed for microwave fields, and the reactor automatically regulated the microwave power to maintain the preset temperature with a control accuracy of ±1 °C. The system was raised to the desired temperature (50–90 °C) and held for a specified reaction time (30–90 min). After completion of the reaction, the slurry was vacuum-filtered, and the elemental concentrations in the leachate were determined using inductively coupled plasma optical emission spectroscopy (ICP-OES). The leaching efficiency of each element was calculated according to Equation (8):

(8) $$E_{i}=\frac{c_{i}\times V}{m\times w_{i}}\times 100\%,$$

where

*E_i_*—extraction rate of element *i* (%);
*c_i_*—measured concentration of element *i* in solution (g·L^−1^);
*V*—bulk volume of the leaching solution (L);
*m*—initial mass of the solid specimen (g);
*w_i_*—the mass fraction of element *i* in the raw solid (dimensionless).

(2) Countercurrent leaching process

The leachate obtained under the optimal microwave leaching conditions was directly employed as the acidic medium and reacted with 5 g fresh pre-decalcified slag under identical optimal conditions. Following each leaching cycle, the solid–liquid mixture was separated by vacuum filtration. The leachate was retained for the subsequent stage, while the solid residue underwent collection and drying. The recovered leachate was repeatedly used to treat fresh pre-decalcified slag in a stepwise manner under the same operating conditions. After each stage, the V concentration was measured until the enrichment level reached a steady state. The number of repetitions was defined as the number of countercurrent leaching stages.

**Figure 23:**
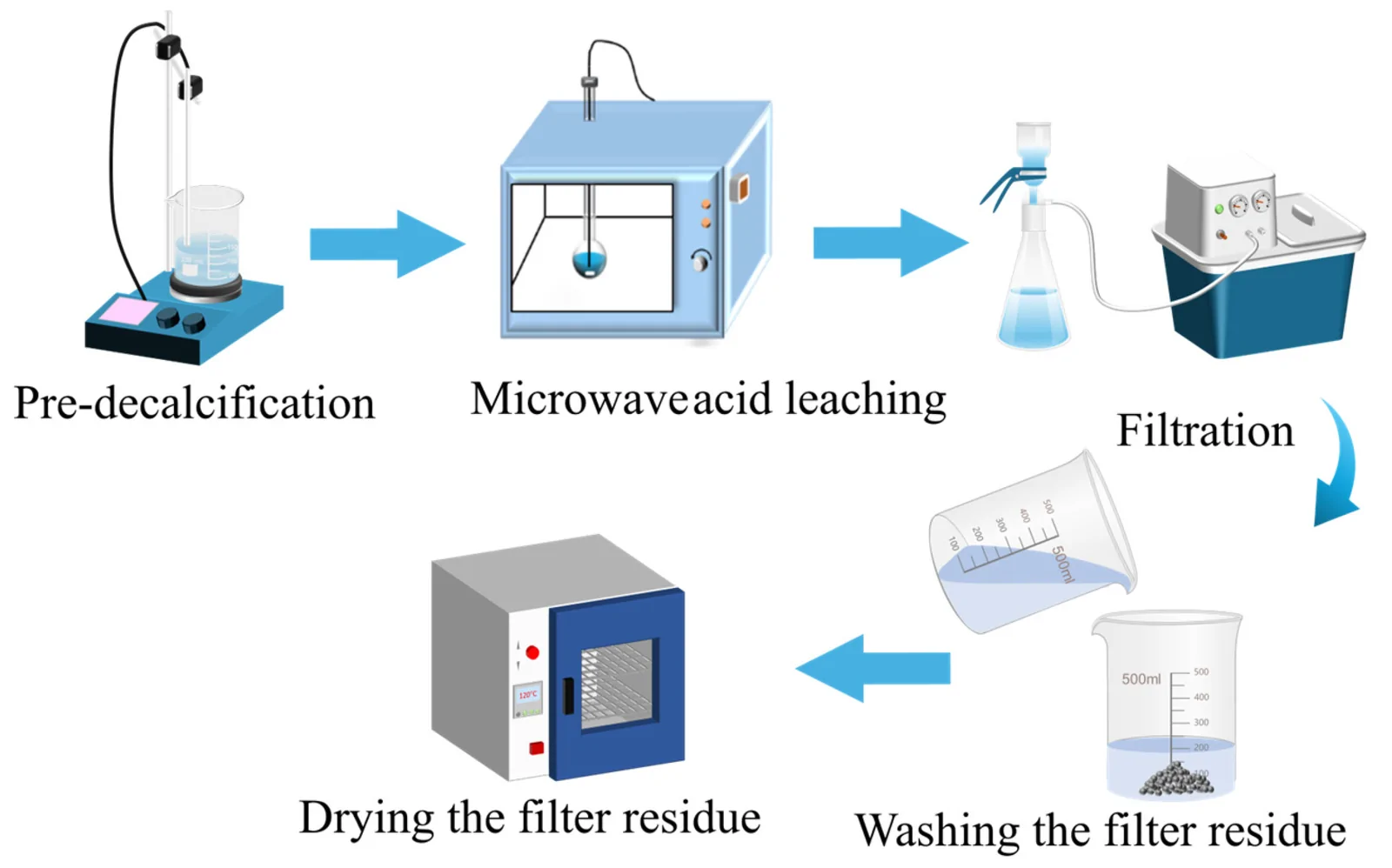
Schematic diagram of the experimental procedure.

The overall experimental procedure is illustrated in Figure 23.

## 4. Conclusions

1. The effects of sulfuric acid concentration, leaching temperature, leaching time, and L/S ratio on vanadium leaching behavior were systematically investigated. Drawing upon single-factor experimental results, the optimal conditions under microwave heating were established as 70 °C, 60 min, an L/S ratio of 8 mL·g^−1^, and 15 wt.% sulfuric acid concentration, under which a vanadium leaching rate of 74.43% was achieved. After two-stage leachate reuse, the normalized V enrichment index increased from 74.43% to 89.93%, while further extension to the third stage provided only a limited additional enrichment.
2. XRD and SEM-EDS characterization revealed that the leaching residue was mainly composed of calcium sulfate hemihydrate, exhibiting a compact acicular morphology with partial agglomeration of fine particles. Such a structure may encapsulate unreacted raw material, thereby hindering further vanadium leaching.
3. Kinetic analysis revealed that vanadium dissolution conformed to a constant-particle-size shrinking-core model and was predominantly controlled by internal diffusion within the first 60 min, while the contribution of the interfacial chemical reaction could not be neglected. The apparent activation energy was estimated at 57.20 kJ·mol^−1^. Furthermore, combining the concentration dependence of the reaction rate, the apparent reaction order was 1.80, and the semi-empirical kinetic expression was established as ln*k_d_* = 1.80ln*C* − 11.18.

## Figures and Tables

**Table 1:** Leaching rates of V and major impurity elements under the optimal conditions.

Element	V	Mg	Fe	Ca	P	Si
Leaching rate (%)	74.43	46.20	34.83	30.64	25.72	8.98

**Table 2:** Main chemical compositions of vanadium-bearing steel slag and pre-decalcified slag (wt.%).

Chemical Composition	CaO	Fe_2_O_3_	MgO	Al_2_O_3_	V_2_O_5_	P_2_O_5_	MnO	TiO_2_	SiO_2_	Cr_2_O_3_	Other	Total
Vanadium-bearing steel slag	40.83	30.31	10.69	2.48	2.19	2.15	1.85	1.66	1.25	0.69	5.90	100
Pre-decalcified slag	19.87	34.45	11.41	2.98	2.49	3.26	1.81	2.94	1.91	0.93	17.95	100

## Data Availability

The raw data supporting the conclusions of this article will be made available by the authors on request.

## Abbreviations

The following abbreviations are used in this manuscript:

XRD	X-ray Diffraction
SEM-EDS	Scanning Electron Microscope-Energy Dispersive X-ray Spectroscopy
ICP	Inductively Coupled Plasma Spectroscopy
L/S ratio	Liquid-to-solid ratio
XPS	X-ray Photoelectron Spectroscopy
